# Building eco-surplus culture among urban residents as a novel strategy to improve finance for conservation in protected areas

**DOI:** 10.1057/s41599-022-01441-9

**Published:** 2022-11-29

**Authors:** Minh-Hoang Nguyen, Thomas E. Jones

**Affiliations:** 1 Graduate School of Asia Pacific Studies, Ritsumeikan Asia Pacific University, Beppu, Oita, 874-8577 Japan; 2 Centre for Interdisciplinary Social Research, Phenikaa University, Yen Nghia Ward, Ha Dong District, Hanoi, 100803 Vietnam

**Keywords:** Environmental studies, Psychology, Finance

## Abstract

The rapidly declining biosphere integrity, representing one of the core planetary boundaries, is alarming. In particular, the global numbers of mammals, birds, fishes, and plants declined by 68% from 1970 to 2016. One of the most widely accepted measures to halt the rate of biodiversity loss is to maintain and expand protected areas that are effectively managed. However, doing so requires substantial finance derived from nature-based tourism, specifically visitors from urban areas. Using the Bayesian Mindsponge Framework (BMF) for conducting analysis on 535 Vietnamese urban residents, the current study examined how their biodiversity loss perceptions can affect their willingness to pay for the entrance fee and conservation in protected areas. We found that perceived environmental degradation, loss of economic growth, loss of nature-based recreation opportunities, and loss of knowledge as consequences of biodiversity loss indirectly affect the willingness to pay through the mediation of the attitude towards conservation. Notably, perceived knowledge loss also has a direct positive influence on the willingness to pay for the entrance fee and conservation. In contrast, perceived loss of health is negatively associated with the attitude towards conservation. Based on these findings, we suggest that building an eco-surplus culture among urban residents by stimulating their subjective cost-benefit judgments towards biodiversity loss can be a promising way to generate more finance from nature-based tourism for conservation in protected areas and ease the domestic government’s and international organizations’ funding allocation problems. Eco-surplus culture is a set of pro-environmental attitudes, values, beliefs, and behaviors shared by a group of people to reduce negative anthropogenic impacts on the environment and conserve and restore nature.

## Introduction

Among nine planetary boundaries, which help define “safe operating space” for human societies development without driving the Earth system away from a Holocene-like condition, climate change and biosphere integrity (measured by the rate of biodiversity loss) are two core boundaries (Steffen et al., 2015). Despite the vital roles of biosphere diversity in the Earth system, the biodiversity loss rate is occurring at an unprecedented rate. Around 1 million species are threatened with extinction, according to the Intergovernmental Science-Policy Platform on Biodiversity and Ecosystem Services (2019). Moreover, the global numbers of mammals, birds, fishes, and plants also dropped by 68% from 1970 to 2016 (World Wildlife Fund, 2020). To curb the substantial degradation of biological diversity, keeping and expanding protected areas are suggested as fundamental solutions.

The past several decades have seen the profound development and expansion of protected areas worldwide in geography and function (Watson et al., 2014). Since the establishment of the world’s first national park—Yellowstone national park—in 1872, the total area of protected areas and other effective area-based conservation measures (OECMs) have covered at least 16.64% (22.5 million km^2^) of land and inland water ecosystems, and 7.74% (28.1 million km^2^) of coastal waters and the ocean (UNEP-WCMC and IUCN, 2021). The areas of particular importance for biodiversity and ecosystem services have been increasingly covered, with 65.5% of Key Biodiversity Areas partially or fully protected (UNEP-WCMC and IUCN, 2021). Along with the geographical expansion, protected areas’ functions have also been diversified to achieve various conservation, social and economic targets (Watson et al., 2014). Due to protected areas’ vital roles, effective management and expansion of protected areas over terrestrial and marine areas are integrated into global agendas. For example, conserving at least 17% of terrestrial and inland water and 10 % of coastal and marine areas, especially those with important biodiversity and ecosystem services, by 2020 was set as Target 11 of the Aichi Biodiversity Targets. Meanwhile, Goals 14 and 15 of the United Nations’ Sustainable Development Goals emphasize the conservation, restoration, and promotion of sustainable use of marine and terrestrial ecosystems, which greatly rely on protected areas (Andriamahefazafy et al., 2022; FAO, 2021; UNEP-WCMC, BLI, IUCN—United Nations Environment Program-World Conservation Monitoring Centre, BirdLife International, and Nature IUftCo, 2020).

Expansion and effective management of protected areas require substantial, sustainable finance. Even though the protected areas are increasingly designated, financial support for protected areas is falling behind, leading to poor management and rampant “paper park” situations, especially in developing countries (Bovarnick et al., 2010; Emerton et al., 2006; Dharmaratne et al., 2000; Thur, 2010). In Vietnam, national parks mostly receive funding from the state for operations and maintenance. According to the Division of Nature Conservation, state funding (channeled through central and local levels of government) contributed up to 78.07% of revenue for national parks in 2015, approximately $8 million (around 175 billion VND) (Pham and Bui, 2020). Conservation management budget may also come from international donors and non-state organizations, such as World Wide Fund for Nature (WWF), Fauna & Flora International (FFI), International Labour Organization (ILO), Vietnam Conservation Fund (VCF), Vietnam Environment Protection Fund (VEPF), etc. (Pham and Bui, 2020). Nonetheless, there remain many constraints. Domestic government subsidies are widespread but insufficient and lack priority, whereas international aids are large but can only focus on large, site-specific projects (Bui et al., 2021). As a result, tourism is endorsed by many scientists as a sustainable financing source for biodiversity conservation in protected areas if it is effectively managed (Jones et al., 2021; Whitelaw et al., 2014).

The demand for nature-based tourism is one of the fundamental reasons driving people to visit protected areas. The revenue generated from the influx of visitors to protected areas is massive. On a global scale, Balmford et al. (2015) estimate that around 8 billion visits are made per year to the world’s terrestrial protected areas. These visits generate roughly $600 billion per year in direct in-country expenditure and $250 billion per year in consumer surplus. Thanks to the income generated by tourism, many national parks (e.g., Hustai National Park in Mongolia and South African National Parks) can pay more than 50% of their expenditure for park operation and conservation of some endangered species (Bovarnick et al., 2010; Buckley, 2012; Leung et al., 2018; South African National Parks, 2016). Moreover, if the benefits of tourism are allocated in fair and equitable ways, tourism development also helps sustain the local livelihood, which reduces the pressure on conservation efforts (Naughton-Treves et al., 2005; Walpole and Goodwin, 2001; World Bank, 2021).

Although nature-based tourism can be a good income source to finance conservation activities, it also negatively affects biodiversity and ecosystems in protected areas. A comprehensive assessment of 1,961 terrestrial protected areas across 149 countries demonstrates that recreational activities are the second most common threat to biodiversity (occurring in 55% of the studied protected areas), just behind unsustainable hunting (occurring in 61% of protected areas) (Schulze et al., 2018). It is reported that tourism-induced activities, construction of infrastructure (e.g., resorts, roads, trails), and introduction of alien species can lead to habitat destruction and biodiversity loss (Kelly et al., 2003; Hasler and Ott, 2008; Luo et al., 2018; Tolvanen and Kangas, 2016). For example, in a case study of the Chinese giant salamander (*Andrias davidianus*), Luo et al. (2018) discovered that high levels of tourism disturbance reduce the habitat quality and species population size by increasing noise, pathogenic microbes, the concentration of nitrogen, and total phosphorus, and mitigating dissolved oxygen in the water (Luo et al., 2018). In a review of tourism’s impact on threatened species in the Pacific, tourism is attributed to the threatened status of 282 species in the region. The adverse impact of tourism on biodiversity is even higher in countries with large tourism industries (Morrison, 2012). Therefore, without proper management, increased tourism activities may threaten the integrity of protected areas (Newsome et al., 2012, 2018).

For proper management of tourism, many aspects need to be fulfilled. One of the essential aspects is to maximize the environmental and economic values attributable to (or achievable through) nature-based tourism (Whitelaw et al., 2014; Eagles and Hillel, 2008). As such, it is necessary to improve the effectiveness of financing from visitors to protected areas. Levying the fee is widely used to generate revenue from visitors within the protected area. Such fees can appear under various forms, like the fee within a tour, entrance fee, conservation fee, user fee, etc. (Thur, 2010; Whitelaw et al., 2014). Visitors’ willingness to pay for the fee is distinct depending on the protected areas’ features and the visitors’ characteristics (Wang and Jia, 2012; Bhandari and Heshmati, 2010; Estifanos et al., 2021; Baral and Dhungana, 2014; Gelcich et al., 2013). For instance, income level, educational attainment, and institutional trust are strong predictors of an increasing willingness to pay for the entrance fee in the Dalai Lake protected area in northeast China (Wang and Jia, 2012). Visitors are more willing to pay more for the protection of Ethiopian wolves if the wolf population increases (Estifanos et al., 2021). However, studies also show that a certain number of visitors are unwilling to pay because they attribute biodiversity conservation to the government’s responsibility (Wang and Jia, 2012; Bhandari and Heshmati, 2010).

Therefore, one question arises: “could we improve the visitors’ willingness to pay for entrance fees and conservation?” We think there is, and it is, to improve the willingness to pay among the growing number of urban residents—potential visitors to protected areas, besides international visitors (Fredman and Tyrväinen, 2010; Lundmark and Müller, 2010; Frost et al., 2014; Jones and Nguyen, 2021). Urban population is a great potential market to finance protected areas and related conservation efforts because urban people have both the desire and the financial capacity for nature-based tourism (Fredman and Tyrväinen, 2010; Lundmark and Müller, 2010; Frost et al., 2014; Jones and Nguyen, 2021).

Nevertheless, how can the willingness to pay among urban residents be improved? We hypothesize that the willingness to pay for the entrance fee and conservation can be improved by building an eco-surplus culture among urban residents. Eco-surplus culture is the term coined by Vuong (2021) to indicate a culture that values the protection and healing of nature. The concept is suggested as the 11th element, complementing Harrison’s (2000) 10 progressive cultural values. To elaborate on the term, we adopt Matsumoto and Juang’s (2016) definition of culture and define eco-surplus culture as a set of pro-environmental attitudes, values, beliefs, and behaviors that are shared by a group of people to reduce negative anthropogenic impacts on environments as well as conserve and restore nature.

In this study, the conservation endorsement attitude can be considered a representative value of the eco-surplus culture. Nguyen and Jones (2021) indicate that perceived consequences of biodiversity loss, such as environmental degradation, losses of economic growth, nature-based recreation opportunities, health, and knowledge, are positively associated with a positive attitude towards the prohibition of wildlife consumption. Therefore, it is also possible that urban residents’ biodiversity loss perceptions are positively associated with their attitude towards conservation and, thus, eco-surplus culture. Further explanations of the relationships between biodiversity loss perceptions, the conservation-related attitude, and willingness to pay are shown in the Model Construction sub-section.

To our knowledge, most of the studies regarding willingness to pay are conducted on-site with visitors visiting the protected areas, and little is known about the willingness to pay among urban residents and its predictors. Thus, the current study employed the Bayesian Mindsponge Framework (BMF) analytics to examine the link between perceptions of biodiversity loss, the attitude towards conservation, and willingness to pay for the entrance fee and conservation in protected areas among 535 residents in Vietnam’s largest cities. The BMF analytics combines Vuong’s (2022a) mindsponge theory as a foundation for model construction and Bayesian inference as an analytical approach to estimate the constructed models.

Vietnam’s urban residents are suitable sampling targets for this study’s objectives because of two main reasons. Vietnam is a Southeast Asian country located in the Indo-Burma region—one of the most biologically important and threatened hotspots worldwide, so it contains a great diversity of species that include more than 13,200 floral species and around 10,000 faunal species (Fauna and Flora International, 2021). Additionally, the rapid urbanization and rising income of urban residents in Vietnam may increase the demand for nature-based tourism (The World Bank, 2022).

## Methods

### Study site and samples

Using the dataset of Nguyen (2021), the current study examined the associations between perceptions towards biodiversity loss, the attitude towards conservation, and willingness to pay for the entrance fee and conservation among Vietnamese urban residents. The dataset was systematically designed and generated through four main steps: (1) questionnaire design, (2) survey collection, (3) data check and validation, and (4) dataset generation.

As there was limited knowledge regarding the perceptions of biodiversity and biodiversity loss in the Asian context in general and Vietnam in particular, in-depth interviews were initially conducted with 38 residents in Ho Chi Minh and Hanoi capital cities from November 15 to December 26, 2020. The participants’ profiles (e.g. gender, age, occupation, etc.) were purposively selected to ensure the diversity of opinions. Nguyen (Nguyen, 2021) also applied the ‘theoretical saturation’ principle to determine when to stop the interview process (Creswell and Poth, 2018). Based on the responses of 38 people, the questionnaire was designed.

From June 18 to August 8, 2021, the questionnaire was distributed through a Web-based survey via Google Forms using the snowball sampling strategy. People living in urban areas were intentionally targeted. The participants were asked to read and agree with a consent form explaining the questionnaire’s contents, purposes, and the confidentiality of respondents. Finally, 581 responses were acquired.

Next, a four-step quality check was performed to remove ineligible samples. To elaborate, respondents with residency in non-urban areas, ages <18, duplicate emails, and poor-quality answers were excluded. After the validation, 535 samples remained. Finally, the dataset was generated and saved under comma-separated value format for later use. The dataset was peer-reviewed by two referees and made available on the open repository for later reproduction, validation, and transparency. More details of the dataset can be found here: 10.11922/sciencedb.j00104.00097.

In this study, we employed eight variables that can be categorized into three main groups. The first group includes five variables demonstrating how urban people perceive the consequences of biodiversity loss in five aspects: (1) environmental degradation, (2) loss of economic growth, (3) loss of nature-based recreation opportunities, (4) loss of health, and (5) loss of knowledge. These five variables were generated from ten variables in the dataset. Some variables are relatively similar, so we grouped them into one variable and took the average value. Specifically, perceived pollution and climate change as consequences of biodiversity loss were grouped into *EnvironmentalDegradation*, with 0.88 of Cronbach alpha; perceived loss of green space, natural esthetics, and nature-based recreation were grouped into *NatureRecreationLoss*, with 0.85 of Cronbach alpha; perceived reduction of physical health, mental health, and life expectancy were grouped into *HealthLoss*, with 0.92 of Cronbach alpha; *EconomicGrowthLoss* and *KnowledgeLoss* remained the same (see Table 1).

**Table 1:** Variable description.

Variable	Meaning	Type of variable	Value
*EnvironmentalDegradation*	Whether the respondent perceives environmental degradation (pollution and climate change) as a consequence of biodiversity loss	Numerical	Ranging from 1 (strongly disagree) to 4 (strongly agree)
*EconomicGrowthLoss*	Whether the respondent perceives the loss of economic growth as a consequence of biodiversity loss	Numerical	Ranging from 1 (strongly disagree) to 4 (strongly agree)
*NatureRecreationLoss*	Whether the respondent perceives the loss of nature-based recreation opportunities (loss of green space, natural esthetics, nature-based recreation) as a consequence of biodiversity loss	Numerical	Ranging from 1 (strongly disagree) to 4 (strongly agree)
*HealthLoss*	Whether the respondent perceives the loss of health (reduction of physical health, mental health, and life expectancy) as a consequence of biodiversity loss	Numerical	Ranging from 1 (strongly disagree) to 4 (strongly agree)
*KnowledgeLoss*	Whether the respondent perceives the loss of knowledge as a consequence of biodiversity loss	Numerical	Ranging from 1 (strongly disagree) to 4 (strongly agree)
*Conservation*	Whether the respondent agrees conservation is a preventive measure of biodiversity loss	Numerical	Ranging from 1 (strongly disagree) to 4 (strongly agree)
*WillingEntranceFee*	Whether the respondent is willing to pay for the entrance fee when visiting protected areas	Binary	Agree = 1 Disagree = 0
*WillingDonation*	Whether the respondent is willing to pay for the entrance fee when visiting protected areas	Binary	Agree = 1 Disagree = 0

The second group only has one variable that indicates the respondents’ attitude towards conservation as a preventive measure of biodiversity loss. The last group consists of two variables implying the willingness to pay for the entrance fee and willingness to donate for conservation if the respondents have a chance to visit protected areas.

### Model construction

The BMF analytics, which combines the mindsponge theory (or mindsponge mechanism)’s ability to explain psychological complexity in the human mind and the statistical advantages of Bayesian, was employed as the method in our study (Nguyen et al., 2022a, 2022b). This analytical approach has been found effective in investigating various psychological phenomena, such as the attitude towards biodiversity loss preventive measures, suicidal ideation, book-reading interest, air-pollution-induced migration intention, etc. (Nguyen and Jones, 2021; Nguyen et al., 2021; Vuong et al., 2021a, 2021b, 2022a). In this study, models were initially constructed based on the mindsponge information processing mechanism to examine how perceptions towards biodiversity loss may affect the willingness to pay for the entrance fee and conservation through the endorsement of conservation as a preventive measure (Vuong, 2023; Vuong and Napier, 2015).

According to the mindsponge mechanism, an individual has a mindset, or a set of core values, that influences thinking, attitudes, and behaviors. For information to enter the mindset, it has to pass through the multi-filtering system. The multi-filtering system consists of two major components: (1) cost–benefit judgments and (2) trust evaluation. These two components determine whether to accept, reject, or keep the information in the buffer zone for later use or assessment. Both the cost–benefit judgments and trust evaluation are based on the preferences of the mindset and information absorbed from the environment (Nguyen et al., 2021; Vuong et al., 2022a). In this study, we employed two fundamental principles of the mindsponge mechanism to formulate hypotheses and construct models (Vuong, 2023; Nguyen et al., 2022b; Vuong et al., 2022a):

- The information processing mechanism within the mind (the multi-filtering process) is based on the trust evaluator and subjective cost–benefit judgment to maximize the perceived benefits and minimize perceived costs.
- The outputs of conscious and subconscious mental processes (e.g., attitudes, thoughts, feelings, behaviors, etc.) are influenced by the values within the mind (mainly by the core values in the mindset).

It is assumed that a person is willing to pay for the entrance fee and donation for conservation when the information related to the willingness to pay for such purposes could enter their mindset and influence the respondent’s answer. Grounded on the mindsponge mechanism’s principles, a condition needs to be satisfied for the information to appear in the mindset: paying for the entrance fee and donation is subjectively perceived as beneficial by the person. Entrance fee and donation payments are usually associated with conservation efforts, so it is expected that the existence of information endorsing conservation in the mindset will attach more beneficial values to the act of paying for the entrance fee and donation, which subsequently leads to a higher probability of being willing to pay.

Applying the same reasoning approach can also explain how the ideation endorsing conservation as a method of preventing biodiversity loss appears in the mindset. Objectively, biodiversity loss can result in multiple negative consequences, such as environmental problems, loss of economic growth, loss of health, loss of nature-based creation opportunities, loss of knowledge, etc. However, a person will be less likely to acquire the ideation of endorsing conservation if their mind is not aware of biodiversity loss’s adverse consequences. In other words, a person needs to subjectively perceive the adverse effects of biodiversity loss to be more likely to accept information associated with preventive measures (here is conservation) to enter their mindset.

**Fig. 1:**
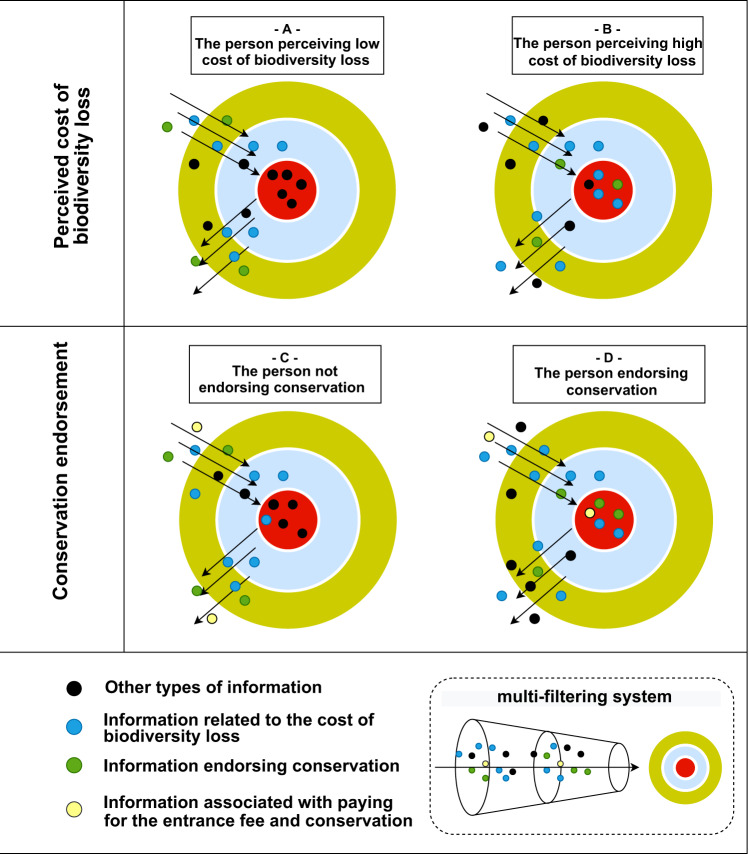
The information-based psychological process leading to conservation endorsement and willingness to pay. The illustration is visualized based on information absorption and ejection processes of the mindsponge mechanism.

Visual elaborations of the information processes are shown in Fig. 1. There are four scenarios:

- In scenario A, there is a low amount of information related to the cost of biodiversity loss in the mindset, so the perceived cost of biodiversity loss is insignificant, making the person less likely to seek and absorb information related to biodiversity loss preventive measures.
- In scenario B, there is a high amount of information related to the cost of biodiversity loss in the mindset, so the perceived cost of biodiversity loss is significant, making the person more likely to consciously or subconsciously seek and accept information related to biodiversity loss preventive measure to enter the mindset. As conservation is a typical preventive measure, the information endorsing conservation as a preventive measure for biodiversity loss is more likely to be sought and accepted to enter the mindset.
- In scenario C, although the person, to some degree, perceives the cost of biodiversity loss, the amount of information related to the cost of biodiversity loss in the mindset is insufficient to influence the information-seeking behaviors and the filtering process to allow conservation endorsement information to enter the mindset. Without the information endorsing conservation in the mindset, the ideation of paying for the entrance fee and conservation donation is less likely to emerge in the mindset and lead to the willingness to pay.
- In scenario D, when the information endorsing conservation as a preventive measure for biodiversity loss emerges in the mindset, it would subsequently affect the information-seeking behaviors and filtering process to accept information involved with conservation to enter the mindset. Among conservation supporting methods, paying the entrance fee and donating for biodiversity conservation in protected areas can be perceived as two common ways. Thus, the information associated with paying for the entrance fee and conservation donation is more likely to be absorbed into the mindset.

From these scenarios, the associations between biodiversity loss perceptions and the attitude toward conservation are expected to be positive. Moreover, it is also expected that urban residents’ biodiversity loss perceptions might positively affect their willingness to pay for the entrance fee and donation for conservation through the mediation of the attitude towards conservation.

To check our assumptions, we constructed the following models. Model 1 examines the associations between perceived consequences of biodiversity loss and support for conservation as a preventive measure among urban residents. Models 2a and 2b estimate the impacts of the urban residents’ support for conservation on their willingness to pay for the entrance fee and conservation, respectively. Finally, Model 3a and 3b were constructed to check whether the associations between biodiversity loss perceptions and willingness to pay are also direct or indirect through the attitude towards the conservation. If the direct associations are not confirmed, our assumptions using an information processing mechanism to explain the phenomena can be deemed trustworthy.

$$\begin{array}{l}{{{\mathbf{Model}}}}\,{{{\mathbf{1}}}}:\,{\rm {Conservation}}\sim {\rm {EnvironmentalDegradation}} \\ + \,{\rm {EconomicGrowthLoss}} + {\rm {NatureRecreationLoss}} \\ + \,{\rm {HealthLoss}} + {\rm {KnowledgeLoss}}\end{array}$$

$${{{\mathbf{Model}}}}\,{{{\mathbf{2a}}}}:{\rm {WillingEntranceFee}}\sim {\rm {Conservation}}$$

$${{{\mathbf{Model}}}}\,{{{\mathbf{2b}}}}:\,{\rm {WillingDonation}}\sim {\rm {Conservation}}$$

$$\begin{array}{l}{{{\mathbf{Model}}}}\,{{{\mathbf{3a}}}}:{\rm {WillingEntranceFee}}\sim {\rm {Conservation}} \\ + \,\,{\rm {EnvironmentalDegradation}} +\, {\rm {EconomicGrowthLoss}}\\ + \,\,{\rm {NatureRecreationLoss}} + {\rm {HealthLoss}} + {\rm {KnowledgeLoss}}\end{array}$$

$$\begin{array}{l}{{{\mathbf{Model}}}}\,{{{\mathbf{3b}}}}:\,{\rm {WillingDonation}}\sim {\rm {Conservation}} \\ + \,\,{\rm {EnvironmentalDegradation}} + {\rm {EconomicGrowthLoss}} \\ + \,\,{\rm {NatureRecreationLoss}} + {\rm {HealthLoss}} + {\rm {KnowledgeLoss}}\end{array}$$

### Analytical approach

The constructed models were then analyzed using Bayesian inference aided by the Hamiltonian Monte Carlo algorithm. Reasons for selecting Bayesian inference as the statistical method are several. First, it fits well with models constructed using the mindsponge mechanism (Nguyen et al., 2022a, 2022b). One of the natural advantages of Bayesian analysis is that it treats all properties (including unknown variables) probabilistically (Csilléry et al., 2010; Gill, 2014). When applied to parsimonious models constructed based on the mindsponge mechanism, Bayesian analysis helps researchers avoid adding control variables and focus entirely on the theoretically selected variables, ensuring the parsimony principle (or Occam’s razor) (Nguyen et al., 2022a, 2022b).

Secondly, the data analyzed in this study were not randomly sampled, and its size was modest. Still, the Bayesian analysis can complement this weakness as “Bayesian statistics is not based on large samples (i.e., the central limit theorem) and hence may produce reasonable results even with small to moderate sample sizes, especially when strong and defensible prior knowledge is available” (Depaoli and Van de Schoot, 2017). The mindsponge framework can defend prior selection in the current study against criticism of subjectivity (Nguyen et al., 2022b; Vuong et al., 2022a). At the same time, the Bayesian inference aided by the Hamiltonian Monte Carlo algorithm helps model estimation get rid of the dependence on the asymptotic assumption (Block and Wagner, 2014; Hahn and Doh, 2006).

Another advantage of Bayesian inference is its ability to deal with multicollinearity. In the constructed models, variables of biodiversity loss perceptions may have high correlation levels, possibly leading to multicollinearity. The problem can be solved by alleviating weak data identification problems if priors are incorporated into model fitting (Leamer, 1973; Adepoju and Ojo, 2018; Jaya et al., 2019). Here, we set prior distributions of parameters as a normal distribution with the mean value at 1 and standard deviation at 0.5, representing our beliefs that all studied associations are positive. Besides, the prior-tweaking technique can also be employed to test the sensitivity of the posterior distributions if prior beliefs are changed (Vuong et al., 2021c). If the posteriors only change slightly when we use norm (0,0.5) as priors representing our disbelief in the associations, the results can be deemed robust.

All the Bayesian linear regression analyses were conducted using the bayesvl R package (La and Vuong, 2019). The package offers researchers a user-friendly and intuitive protocol, the ability to visualize beautiful graphics, and cost-effectiveness (Vuong et al., 2020, 2022b). Model fitting was conducted with four Markov chains with 5000 iterations for each chain. The first 2000 iterations were installed as a warmup period. After the simulation, the models’ goodness-of-fit with the data at hand was validated using the Pareto smoothed importance-sampling leave-one-out cross-validation (PSIS-LOO) (Vehtari and Gabry, 2019; Vehtari et al., 2017). If the model fits well with the data, we will continue checking whether the Markov property or the Markov chains’ convergence was held after the simulation process. Effective sample size (*n_eff*) and Gelman-Rubin shrink factor (*Rhat*) are two diagnostic statistics of the Markov chains’ convergence. The convergence can also be diagnosed visually using the trace, Gelman–Rubin–Brooks, and autocorrelation plots.

All the codes and data of this study are deposited on an online repository for future validation and reproduction: https://osf.io/au3hj/.

## Results

The Bayesian linear regression analysis was conducted on 535 Vietnamese urban residents to examine five models proposed in the Model Construction subsection. The estimated results are presented in this section. More than half of the respondents were female (57.08%) and obtained an undergraduate degree as the highest educational level (61.68%). Most of the respondents belonged to the age group ranging from 23 to 40 (47.11%). 85.63% of the participants reported spending most of their lifetime in urban areas, while the percentages of suburban and rural areas were 10.38% and 3.79%, respectively. Regarding the willingness to pay, 97.57% of the respondents were willing to pay for the entrance fee, and 94.95% were willing to donate to conservation projects when visiting a protected area in the future.

### Model 1: The associations between biodiversity loss perceptions and conservation endorsement attitude

**Fig. 2:**
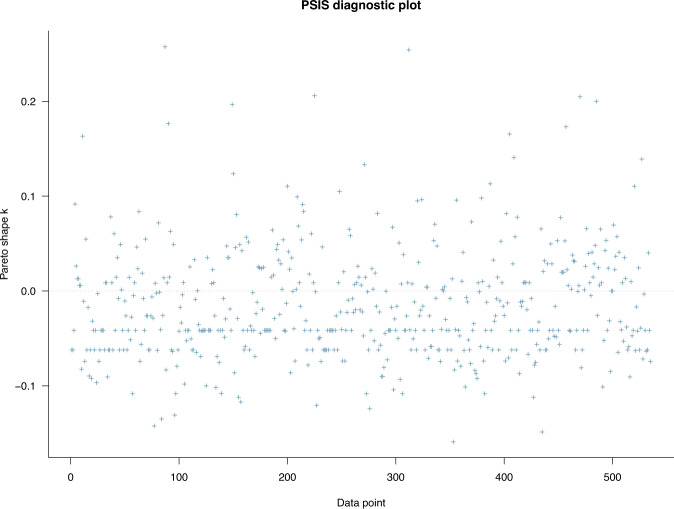
Model 1’s PSIS-LOO diagnosis with priors as norm (1,0.5). The model’s goodness-of-fit can be classified into four levels: (1) ‘good’ if its *k*-values are all below 0.5, (2) ‘OK’ if its *k*-values are more than 0.5 and below 0.7, (3) ‘bad’ if its *k*-values are more than 0.7 and below 1, and (4) ‘very bad’ if its *k*-values are more than 1.

Model 1 was estimated to examine the associations between biodiversity loss perceptions and the conservation-related attitude among urban residents. Five predictor variables used in the model correspond with five different perceptions on the consequences of biodiversity loss: environmental degradation, loss of economic growth, loss of nature-based recreation opportunities, loss of health, and loss of knowledge. The PSIS-LOO test was initially performed to check whether Model 1 fits well with the collected data. The evaluation was based on the estimated *k* of generalized Pareto distribution. According to Vehtari et al. (2017), if the *k*-value is larger than 0.7, it signals model misspecification. If all *k*-values are <0.5, the model has a good fit with the data at hand. All the *k*-values in Fig. 2 are below the 0.5 thresholds, so the model can be considered fit with the data.

Next, it is necessary to verify the convergence of the model using two diagnostic values: effective sample size (*n_eff*) and Gelman–Rubin shrink factor (*Rhat*). The *n_eff* value indicates the number of iterative samples that are not autocorrelated during the stochastic simulation process. Generally, it is accepted that if the *n_eff* value is >1000, the Markov chains are convergent, and the effective samples are sufficient for accurate inference (Vuong et al., 2020). In terms of the *Rhat* value, if the value is much >1, it implies that the chains have not converged, so inference should not be made with the current iterative samples. On the contrary, if the value is equal to 1, it is a good convergence signal (Gelman and Rubin, 1992). As the parameters’ *n_eff* values are all larger than 9000 and *Rhat* values are equal to 1, Model 1 seems to have good convergence, even when prior distributions are different (Table 2).

**Table 2:** Model 1’s simulated posterior results.

Parameters	Informative priors (belief on effect)				Informative priors (disbelief on effect)			
	Mean	SD	*n_eff*	*Rhat*	Mean	SD	*n_eff*	*Rhat*
*Constant*	1.20	0.14	12,522	1	1.22	0.14	12,512	1
*EnvironmentalDegradation*	0.35	0.05	10,215	1	0.35	0.05	12,151	1
*EconomicGrowthLoss*	0.05	0.04	11,215	1	0.05	0.04	11,512	1
*NatureRecreationLoss*	0.18	0.07	9212	1	0.18	0.07	10,215	1
*HealthLoss*	−0.05	0.05	11,215	1	−0.05	0.05	12,562	1
*KnowledgeLoss*	0.13	0.04	12,841	1	0.13	0.04	12,354	1

**Fig. 3:**
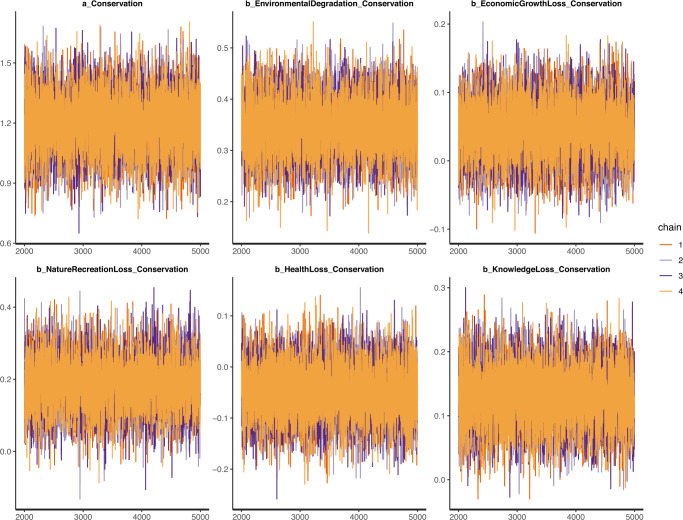
Model 1’s trace plots with priors as norm (1,0.5). The Markov chains are deemed well-convergent if the chains are good-mixing and stationary around an equilibrium.

The trace, Gelman–Rubin–Brooks, and autocorrelation plots validate the convergence again. The trace plot illustrates the MCMC sample values after each successive iteration along the chain. The *y*-axis demonstrates the coefficient’s value, while the *x*-axis demonstrates the number of iterations of the Markov process. The Markov chains can be deemed convergent if the chains are good-mixing (illustrated by the rapid zig-zag motion of each line) and stationary around an equilibrium (the chains stay within the posterior distribution) (McElreath, 2018). Figure 3 demonstrates the trace plots of Model 1, which indicate that the Markov chains are all convergent. It should be noted that, in Fig. 3, all iterations before the 2000^th^ order are removed since warmup iterations are not used for inference.

**Fig. 4:**
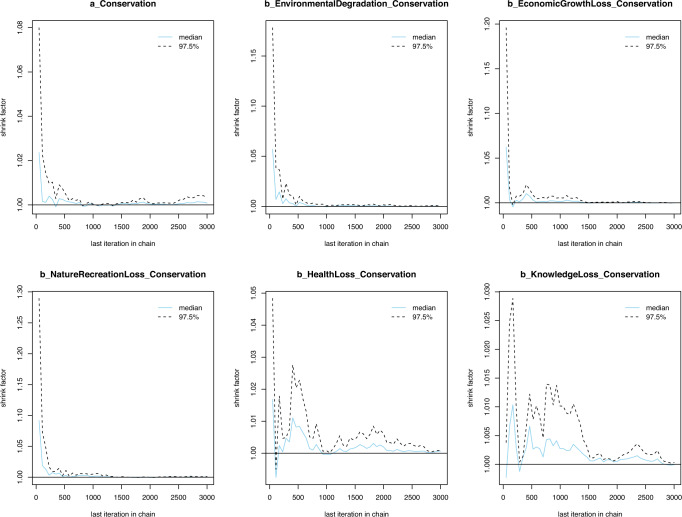
Model 1’s Gelman–Rubin–Brooks plots with priors as norm (1,0.5). The iterative samples are deemed well-convergent if the Gelman–Rubin shrink factor drops rapidly to 1 before the warmup period completes (or before the 2000^th^ iteration).

The Gelman–Rubin shrink factor (*Rhat*) provides a measure of sampling efficiency/effectiveness, which can be visualized on a Gelman–Rubin–Brooks plot. The Gelman–Rubin–Brooks plot is used to see if the shrink factor drops rapidly to 1 before the warmup period is over. If the factor drops to one before the 2000^th^ iteration (warmup period), the simulated samples are said to be convergent (Brooks and Gelman, 1998). As shown in Fig. 4, the shrink factor values drop rapidly to 1 before the warmup period ends. This signals a good convergence of Model 1.

**Fig. 5:**
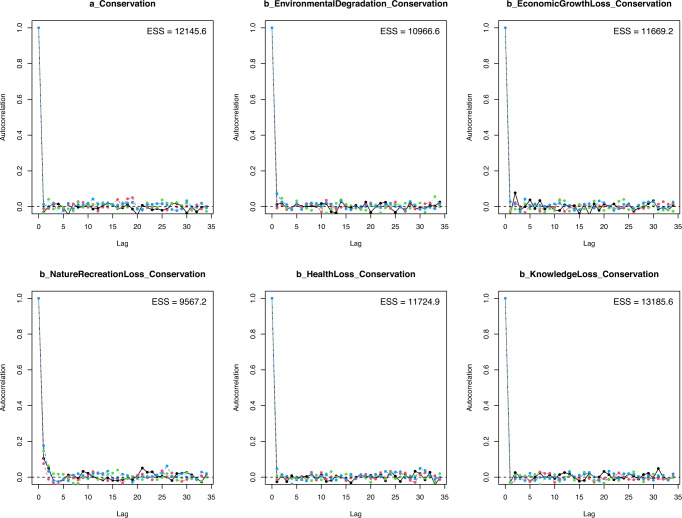
Model 1’s autocorrelation plots with priors as norm (1,0.5). If the autocorrelation levels among iterative samples drop to 0 after some finite lags, the iterative samples are considered independent, and the Markov chains are deemed convergent.

The last diagnostic plot of model convergence is the autocorrelation plot. The autocorrelation plot illustrates the degree of correlation between MCMC samples separated by different lags. For the simulation to generate unbiased estimates of parameters, the MCMC samples should be independent, or the autocorrelation level should be 0 (McElreath, 2018). The autocorrelation plots in Fig. 5 display a rapid decline of the autocorrelation level to 0 after a finite lag, validating that the model’s Markov chains are convergent.

The simulated posteriors employing priors as norm (1,0.5) show that four out of five biodiversity loss perceptions are positively associated with the conservation-related attitude, namely: environmental degradation (*μ*_*EnvironmentalDegradtion*_ = 0.35, *σ*_*EnvironmentalDegradtion*_ = 0.05), loss of economic growth (*μ*_*EconomicGrowthLoss*_ = 0.05, *σ*_*EconomicGrowthLoss*_ = 0.04), loss of nature-based recreation opportunity (*μ*_*NatureRecreationLoss*_ = 0.18, *σ*_*NatureRecreationLoss*_ = 0.07), and loss of knowledge (*μ*_*KnowldegeLoss*_ = 0.13, *σ*_*KnowldegeLoss*_ = 0.04). Interestingly, perceiving loss of health as a consequence of biodiversity loss has the opposite effect on the conservation-related attitude (*μ*_*HealthLoss*_ = −0.05, *σ*_*HealthLoss*_ = 0.05).

**Fig. 6:**
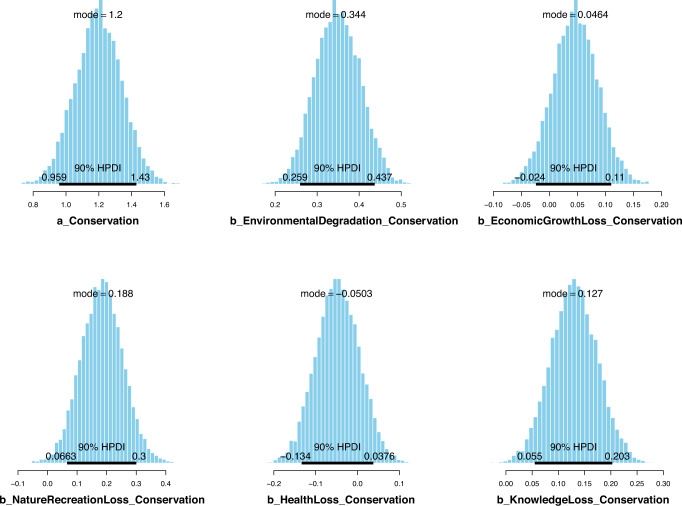
Model 1’s posterior distributions with priors as norm (1,0.5). The plot shows the probability distributions of the parameters with the highest probability range located in the middle. The thick black line at the bottom of the illustration demonstrates the HPDI at 90% of the distribution.

The parameters’ posterior distributions are shown in Fig. 6, along with their Highest Posterior Density Interval (HPDI) at 90%. Apparently, all the credible intervals of *EnvironmentalDegradation*, *EconomicGrowthLoss*, *NatureRecreationLoss*, and *KnowledgeLoss* fall entirely on the positive side of the *x*-axis, suggesting that the positive associations between these variables and outcome variable (*Conservation*) are highly reliable. Regarding *HealthLoss*’s posterior distribution, a majority of its HPDI is located on the negative side but not entirely, and its standard deviation (SD) is equal to the absolute value of the mean, so *HealthLoss*’s negative effect on conservation-related attitude can be deemed moderately reliable. When “prior-tweaking” is performed using the priors representing our disbelief in the associations between biodiversity loss perceptions and the conservation-related attitude, the change is negligible, showing the model’s results are robust (see Table 2).

### Models 2a and 2b: The associations between conservation endorsement attitude and the willingness to pay

**Fig. 7:**
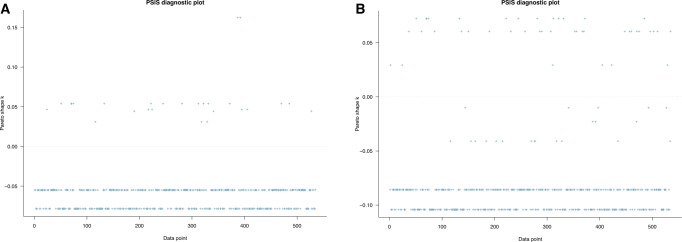
PSIS-LOO diagnosis. Diagnosis for **A** Model 2a and **B** Model 2b with priors as norm (1,0.5). The model’s goodness-of-fit can be classified into four levels: (1) ‘good’ if its *k*-values are all below 0.5, (2) ‘OK’ if its *k*-values are more than 0.5 and below 0.7, (3) ‘bad’ if its *k*-values are more than 0.7 and below 1, and (4) ‘very bad’ if its *k*-values are more than 1.

Models 2a and 2b were examined to check whether urban residents’ conservation-related attitude has a positive impact on their willingness to pay for the entrance fee and conservation when visiting protected areas in the future. The visual PSIS-LOO diagnoses of Models 2a and 2b are displayed in Fig. 7A and B, respectively. *k*-values in both figures are below the 0.5 thresholds, so Models 2a and 2b fit well with the data.

Convergence diagnostic values (n_eff and Rhat) of both models indicate that the models’ Markov chains are convergent. The trace, Gelman-Rubin-Brooks, and autocorrelation plots also confirm the model convergence. Figures A1–A3 are the trace, Gelman–Rubin-Brooks, and autocorrelation plots of Model 2a, respectively, while those of Model 2b are presented in Figs. A4–A6 (see Supplementary).

**Fig. 8:**
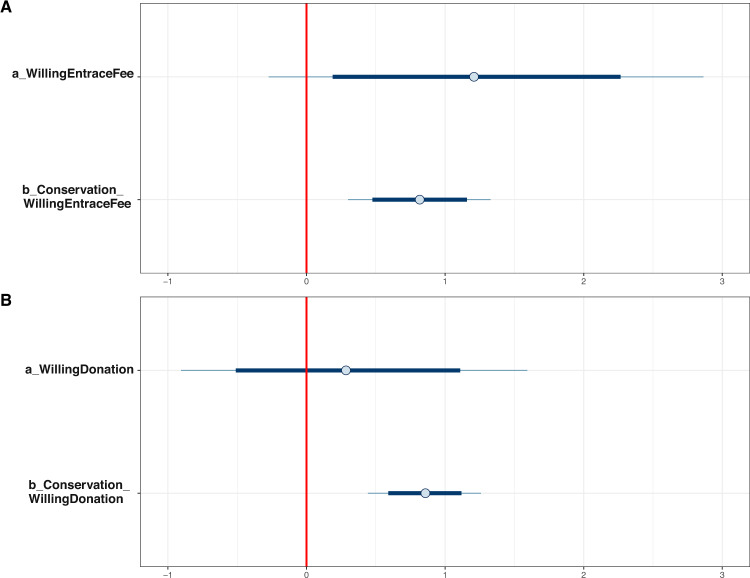
Interval plots of posterior distributions. **A** Model 2a and **B** Model 2b. The red straight line at the origin signifies the boundary between the negative and positive areas. The thick blue lines represent the probability mass within the 89% highest posterior density intervals, while the thin blue lines represent the probability mass located outside the highest credible region.

As can be seen from Table 3, people agreeing that conservation is a preventive measure of biodiversity loss are more willing to pay for entrance fee (*μ*_*Conservation_WillingEntranceFee*_ = 0.81, *σ*_*Conservation_WillingEntranceFee*_ = 0.26) and conservation donation (*μ*_*Conservation_WillingEntranceDonation*_ = 0.86, *σ*_*Conservation_WillingEntranceDonation*_ = 0.21). The posterior distributions of the parameters representing the association between conservation-related attitude and willingness to pay for the entrance fee and conservation are displayed in Fig. 8A and B, respectively. The distributions clearly lie on the positive side of the *x*-axis (separated by the red vertical line), stipulating highly reliable positive associations. When estimating Models 2a and 2b with priors as norm (0,0.5), the posterior distribution’s magnitude declines, but its reliability is still high (see Table 3).

**Table 3:** Model 2a’s and Model 2b’s simulated posterior results.

Parameters	Informative priors (belief on effect)				Informative priors (disbelief on effect)			
	Mean	SD	*n_eff*	*Rhat*	Mean	SD	*n_eff*	*Rhat*
*Model 2a*: *WillingEntraceFee* *~* *Conservation*								
*Constant*	1.23	0.80	2648	1	2.04	0.89	2542	1
*Conservation*	0.81	0.26	2643	1	0.53	0.28	2342	1
*Model 2b*: *WillingDonation* *~* *Conservation*								
*Constant*	0.28	0.63	2698	1	0.76	0.66	2324	1
*Conservation*	0.86	0.21	2517	1	0.70	0.21	2321	1

### Models 3a and 3b: The associations between biodiversity loss perceptions, conservation endorsement attitude, and willingness to pay

**Fig. 9:**
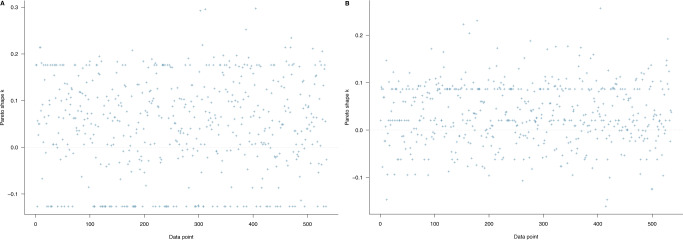
PSIS-LOO diagnosis. PSIS-LOO diagnosis for **A** Model 3a and **B** Model 3b with priors as norm (1,0.5). The model’s goodness-of-fit can be classified into four levels: (1) ‘good’ if its *k*-values are all below 0.5, (2) ‘OK’ if its *k*-values are more than 0.5 and below 0.7, (3) ‘bad’ if its *k*-values are more than 0.7 and below 1, and (4) ‘very bad’ if its *k*-values are more than 1.

Fitting Models 3a and 3b, we aimed to examine the predictions of the conservation-related attitude and biodiversity loss perceptions against the willingness to pay for the entrance fee and conservation in protected areas. The fitting and validating procedures are similar to those employed with Model 1. First of all, PSIS-LOO diagnosis was conducted with both models. The visualizations of *k*-values (all *k*-values are <0.5) in Fig. 9A and B show that Models 3a and 3b are neither under fitted nor overfit with the data.

The *n_eff* and *Rhat* values presented in Table 4 confirm the convergence of Models 3a and 3b (*n_eff* > 8000 and *Rhat* = 1). The visual diagnoses by trace, Gelman-Rubin-Brooks, and autocorrelation plots also verify the convergence. Figures A7–A9 demonstrate Model 3a’s trace, Gelman–Rubin–Brooks, and autocorrelation plots, while Figs. A10–A12 are Model 3b’s trace, Gelman–Rubin–Brooks, and autocorrelation plots, respectively.

**Table 4:** Model 3a’s and Model 3b’s simulated posterior results.

Parameters	Informative priors (belief on effect)				Informative priors (disbelief on effect)			
	Mean	SD	*n_eff*	*Rhat*	Mean	SD	*n_eff*	*Rhat*
*Model 3a*: *WillingEntraceFee* *~* *Conservation* *+* *EnvironmentalDegradation* *+* *EconomicGrowthLoss* *+* *NatureRecreationLoss* *+* *HealthLoss* *+* *KnowledgeLoss*								
*Constant*	0.06	0.96	9412	1	1.49	1.14	8421	1
*Conservation*	0.51	0.31	9778	1	0.39	0.31	10,332	1
*EnvironmentalDegradation*	−0.09	0.35	9321	1	−0.15	0.35	9221	1
*EconomicGrowthLoss*	0.15	0.33	10,654	1	0.03	0.33	10,963	1
*NatureRecreationLoss*	0.22	0.40	10,596	1	0.13	0.39	9654	1
*HealthLoss*	−0.09	0.37	9632	1	-0.18	0.37	9231	1
*KnowledgeLoss*	0.61	0.32	10212	1	0.55	0.31	10321	1
*Model 3b*: *WillingDonation* *~* *Conservation* *+* *EnvironmentalDegradation* *+* *EconomicGrowthLoss* *+* *NatureRecreationLoss* *+* *HealthLoss* *+* *KnowledgeLoss*								
*Constant*	−0.47	0.63	10,512	1	0.37	0.84	10,393	1
*Conservation*	0.64	0.21	10,517	1	0.57	0.25	10,417	1
*EnvironmentalDegradation*	0.18	0.30	9232	1	0.14	0.30	10,963	1
*EconomicGrowthLoss*	−0.02	0.27	10,351	1	−0.09	0.26	11,736	1
*NatureRecreationLoss*	−0.09	0.35	11,542	1	−0.13	0.35	9551	1
*HealthLoss*	0.07	0.31	8021	1	0.02	0.31	10,789	1
*KnowledgeLoss*	0.35	0.27	10,123	1	0.32	0.27	10,545	1

The simulated posterior results of Models 3a and 3b show that the positive associations between conservation-related attitude and willingness to pay for the entrance fee and conservation remain robust with Models 2a’s and 2b’s results. Most biodiversity loss perceptions’ effects on the willingness to pay for the entrance fee and conservation are negligible and unreliable. In particular, their standard deviation values are much higher than the means’ absolute values. Only *KnowledgeLoss* has positive effects on the willingness to pay for the entrance fee (*μ*_*KnowledgeLoss_WillingEntranceFee*_ = 0.61, *σ*_*KnowledgeLoss_WillingEntranceFee*_ = 0.32) and conservation (*μ*_*KnowledgeLoss_WillingDonation*_ = 0.35, *σ*_*KnowledgeLoss_WillingDonation*_ = 0.27).

**Fig. 10:**
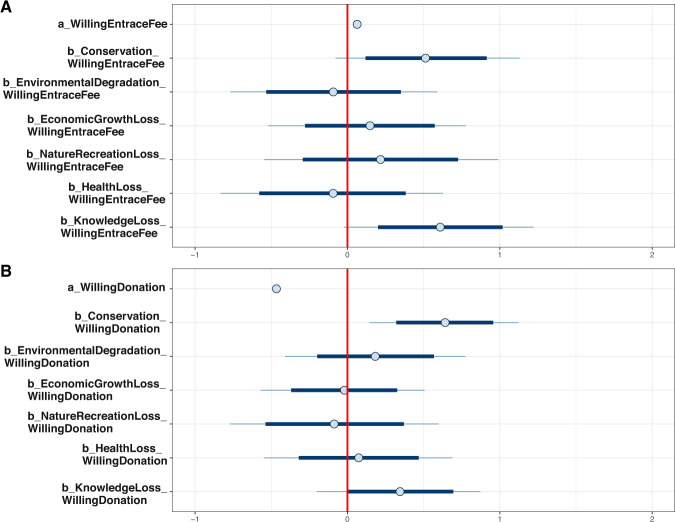
Interval plots of posterior distributions. **A** Model 3a and **B** Model 3b. The thick blue lines represent the probability mass within the 89% highest posterior density intervals, while the thin blue lines represent the probability mass located outside the highest credible region.

The interval plots of Models 3a’s and 3b’s posterior distributions manifest that *Conservation*’s and *KnowledgeLoss*’s HPDIs at 90% are entirely located on the positive side, highlighting the high reliability of their effects on willingness to pay (see Fig. 10A and B, respectively). The HPDI at 90% is illustrated by the thick part in the middle of an interval. After conducting the “prior-tweaking” technique, the parameters’ magnitudes slightly change, but their tendencies are not. Hence, the simulated results are robust.

Based on the results reported above, it is conclusive that biodiversity loss perceptions (*EnvironmentalDegradation*, *EconomicGrowthLoss*, *NatureRecreationLoss*, and *KnowledgeLoss*) have direct positive impacts on the conservation-related attitude and indirect positive impacts on willingness to pay for the entrance fee and conservation through affecting the conservation-related attitude. Meanwhile, perceiving the loss of knowledge as a consequence of biodiversity loss directly positively influences the conservation-related attitude and willingness to pay.

## Discussion

The current study is one of the first studies examining how the urban residents’ biodiversity loss perceptions are associated with their conservation-related attitude and willingness to pay for the entrance fee and conservation in protected areas. The analysis was performed using the BMF analytics on 535 urban residents across Vietnam. Overall, there are three main findings: (1) most biodiversity loss perceptions (*EnvironmentalDegradation*, *EconomicGrowthLoss*, *NatureRecreationLoss*, and *KnowledgeLoss*) have direct positive impacts on conservation-related attitude and indirect impacts on willingness to pay, (2) perceiving loss of health as a consequence of biodiversity loss has negative influence the conservation-related attitude, and (3) perceiving loss of knowledge as a consequence of biodiversity loss has a direct positive influence on conservation-related attitude and indirect positive influences on willingness to pay for entrance fee and conservation.

Evidence from this study suggests that there can be a novel way to improve protected areas financing actively. It is to build an eco-surplus culture among potential visitors to protected areas (specifically, urban residents) by making them aware of the consequences of biodiversity loss.

As shown in this study, the perceived consequences of biodiversity loss can have direct positive impacts on the conservation-related attitude and indirect positive impacts on the willingness to pay. Thus, improving the accessibility of urban residents to information regarding biodiversity and biodiversity loss is vital for building an eco-surplus culture and increasing the aggregate pool of finance for protected areas in the region. Without accessibility to biodiversity-related information, urban residents cannot know that biodiversity loss problems exist, no matter how crucial and severe it is to their lives objectively. Social marketing and demarketing programs, public awareness-raising campaigns, educational activities, and pro-environmental entertaining platforms (e.g., commercial games) are potential methods to create “touchpoints” between urban residents and biodiversity-related information (Vuong et al., 2021c; Haq et al., 2013; Veríssimo, 2019; Veríssimo et al., 2018; Vuong, 2022). In addition, expanding our knowledge of the effects of messages’ content, narrative, and design on changing perceptions, attitudes, and behaviors is also crucial to build an eco-surplus culture effectively. However, Ryan et al. (2020) stipulate that biodiversity conservation marketing is still a nascent field with only 28 studies. Therefore, it is a promising direction for further investigation.

Building an eco-surplus culture is a plausible way to ease the funding allocation problems faced by the domestic government (e.g., widespread but insufficient budget allocation, lack of priority) and international organizations (e.g., large but site-specific funding) (Bovarnick et al., 2010; Bui et al., 2021). By financing social marketing and demarketing programs, public awareness-raising campaigns, educational activities, and pro-environmental entertaining platforms (e.g., commercial games), the government and international organizations can increase the aggregate pool of money that visitors are willing to pay at a regional scale, which indirectly generates finance for protected areas in the region.

To elaborate, assuming that 5000 urban residents visit protected areas nearby the city every month. Before implementing pro-eco-culture campaigns and activities, 60% of them are willing to pay for the entrance fee and conservation initiatives, generating $60,000 a month for protected areas in the region aggregately (each person pays $20). It should be noted that $20 per person is only an assumed number. After implementing pro-eco-culture campaigns and activities, 80% are willing to pay, generating $80,000 ($20,000 surplus) for protected areas in the region. When the aggregate pool of money increases, all protected areas in the region will have a higher chance of benefiting from nature-based tourism (Dharmaratne et al., 2000; Jones et al., 2021).

It is a global trend that urbanization is happening swiftly, and economic power is increasingly concentrated in urban areas, especially large cities (Balsa-Barreiro et al., 2019). The rapid urbanization and economic accumulation of urban people not only affect biodiversity but also rely on it because urbanization is associated with the consumption of natural resources and ecosystem services, including biodiversity (Elmqvist et al., 2013). If biodiversity loss is not halted, not only urbanization and economic growth but also general human development will be catastrophically damaged (Steffen et al., 2015; Díaz et al., 2006). Therefore, building an eco-surplus culture and redirecting the accumulated capital in urban areas to finance the conservation and restoration of biodiversity is essential as it helps create a sustainable cycle of development, in which the net loss of biodiversity can be either balanced or outweighed by the net gain of biodiversity (Aiama et al., 2015). This argument is even more plausible when the shifting demographics, rapid urbanization, exacerbating effects of climate change, increasing diffusion of media technologies, and changing psychological drivers will likely increase the demand for nature-based tourism in Asia-Pacific Region, especially developing countries like Vietnam (Frost et al., 2014). In addition, visitors with better-informed knowledge about the effects of biodiversity and biodiversity loss might have more respect for nature and cause less impact on protected areas (Marion and Reid, 2007).

The effects of biodiversity loss perceptions on the willingness to pay for the entrance fee and conservation validate our assumptions about the role of the individual’s subjective cost-benefit evaluation process in accepting or rejecting information. Furthermore, most of the effects of biodiversity loss perceptions on willingness to pay are indirect (except for the perceived loss of knowledge) and mediated by the attitude towards conservation, showing that the information evaluation process is sequential. In other words, it takes multiple steps for a person to process information and eventually arrive at the ideations and behaviors that benefit them (in this case, it is the willingness to pay for the entrance fee and conservation).

The impact of perceived knowledge loss is relatively special because it influences the willingness to pay both directly and indirectly through the mediation of the conservation-related attitude. Nonetheless, it is unclear why the effect of perceived knowledge is more direct than others, so investigating the link between perceived knowledge loss and support for conservation in general and willingness to pay in particular is a potential direction for later research. Regardless of the causes, the importance of knowledge about nature should be concentrated in public awareness-raising campaigns, social marketing and demarketing programs, and educational activities.

The negative effect of perceived health loss resulting from biodiversity loss on the conservation-related attitude is paradoxical with other biodiversity loss perceptions’ effects. Following the mindsponge thinking, which assumes that people try to maximize their perceived benefits and reduce perceived costs, might help explain this finding partially (Nguyen et al., 2021). In particular, urban residents who perceive health loss as a consequence of biodiversity loss are sensitive to health-related issues. In Vietnam, many perceived “nutritional” and “healthy” traditional medicines are made from wildlife products, such as pangolin scales, tiger bones, bear bile, etc. (Davis et al., 2020, 2019; Sexton et al., 2021). The term “conservation” is usually viewed as a tool for protecting “a subset of biodiversity that includes charismatic species and those on threatened species lists” (Mace et al., 2012), so people sensitive to their health issues might be less likely to support conservation. It should be noted that the explanation here is speculative, so further studies are needed.

Several limitations of this study are presented here for transparency (Vuong, 2020). The convenient sampling strategy due to the prolonged social distancing for COVID-19 containment may lead to selection bias. Thus, the results should be interpreted with precaution. By employing the Bayesian analysis, we could provide precise estimations based on the current dataset, which can be used to compare with studies analyzing random sampling data. Moreover, given the diverse residencies and backgrounds of participants (from cities across Vietnam), we believe our findings are still representative to some extent. Another limitation is that there is no evidence that the willingness to pay before and after arriving at the protected areas will remain the same. Although there are possibilities that urban visitors’ paying willingness decreases due to protected areas’ characteristics and trip features, to some extent, the direct and indirect effects of biodiversity loss perceptions on willingness to pay are still reliable evidence for the notion that improving awareness and knowledge among urban residents can lead to higher willingness to pay in protected areas.

## Supplementary information

Figure A1

Figure A2

Figure A3

Figure A4

Figure A5

Figure A6

Figure A7

Figure A8

Figure A9

Figure A10

Figure A11

Figure A12

## Data Availability

The data that support the findings of this study are peer-reviewed and available on MIT *Data Intelligence* for later replications: https://direct.mit.edu/dint/article/3/4/578/107428/Multifaceted-Interactions-between-Urban-Humans-and. For convenience, all the codes and data of this study are deposited on an online repository for future validation and reproduction: https://osf.io/au3hj/.
