# Supplementary figures and images for: Building eco-surplus culture among urban residents as a novel strategy to improve finance for conservation in protected areas
Source: Humanit Soc Sci Commun. 2022 Nov 29;9(1):426. doi: 10.1057/s41599-022-01441-9 (PMC9708145; doi:10.1057/s41599-022-01441-9)

**a\_WillingEntranceFee**

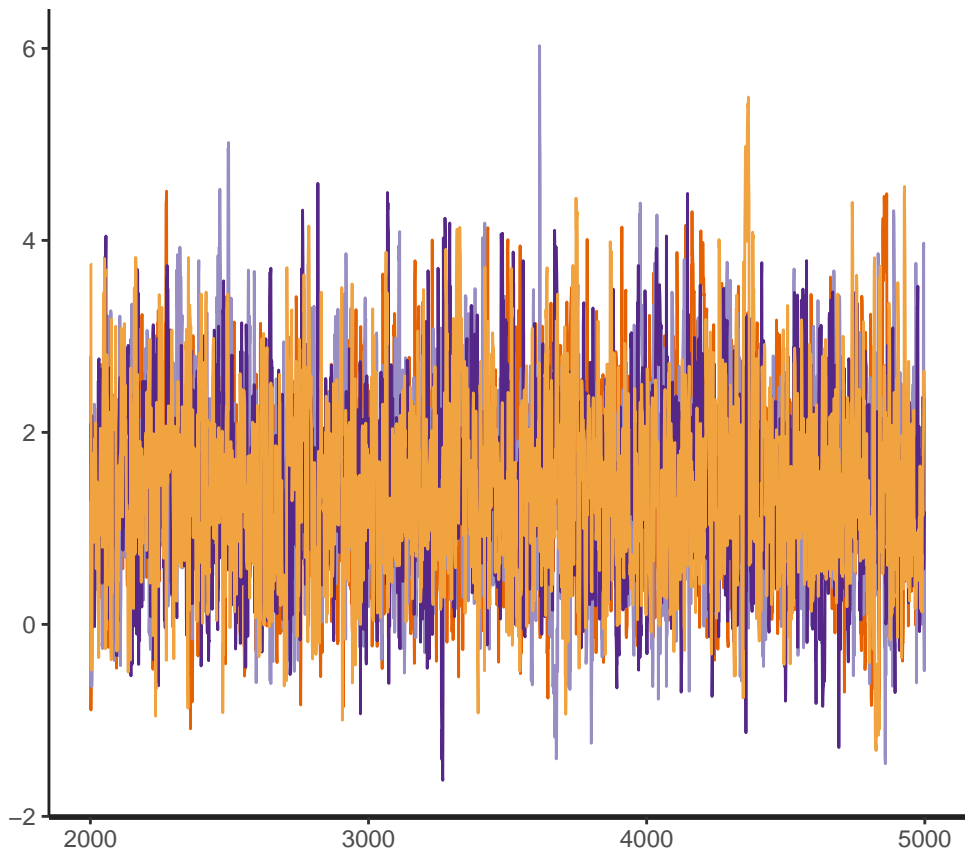

**b\_Conservation\_WillingEntranceFee**

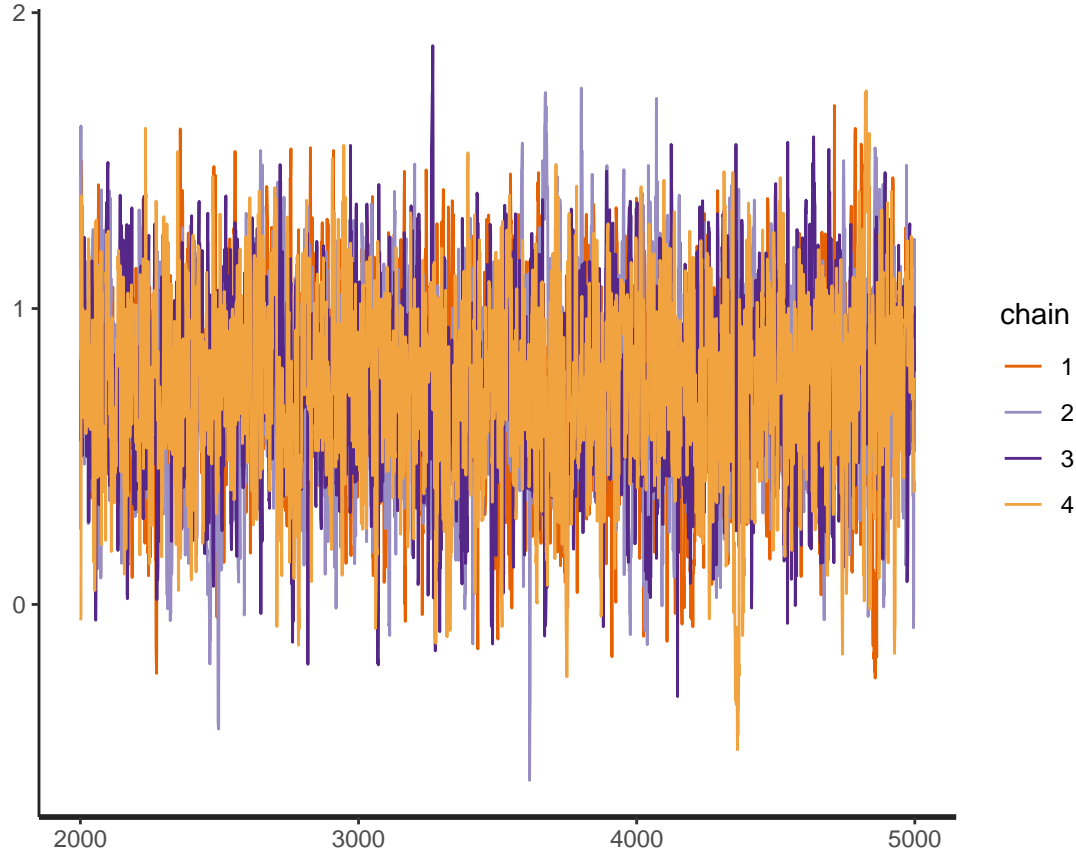

Supplement: Supplementary file 1 — Figure A1 [file 41599_2022_1441_MOESM1_ESM.pdf]

**a\_WillingEntranceFee**

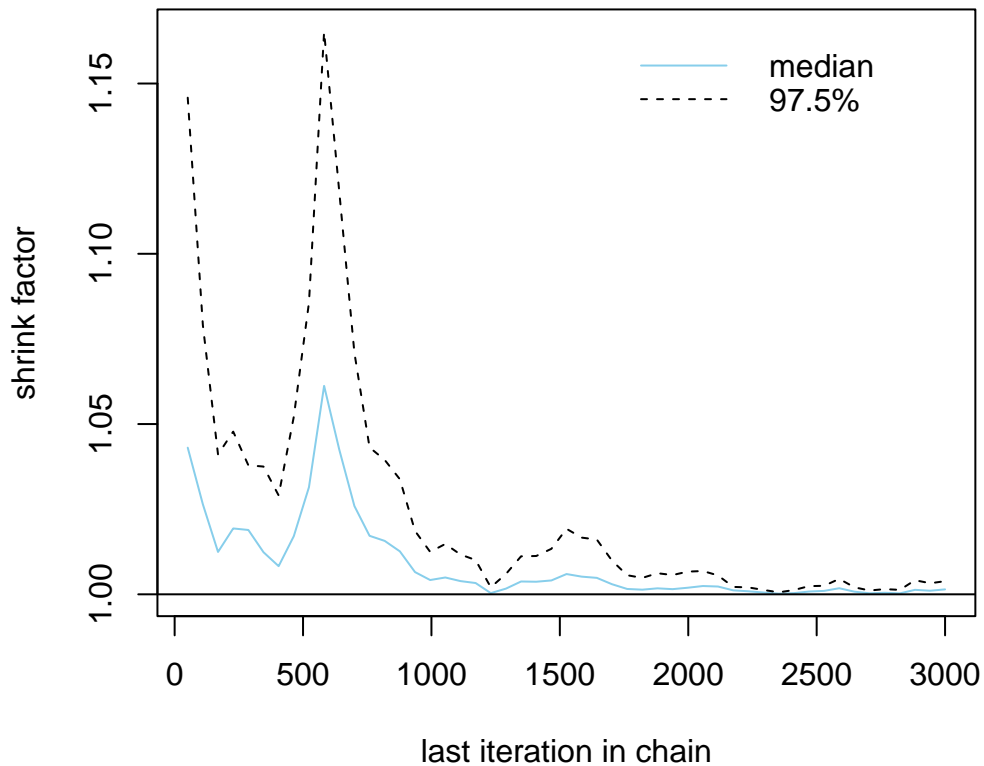

**b\_Conservation\_WillingEntranceFee**

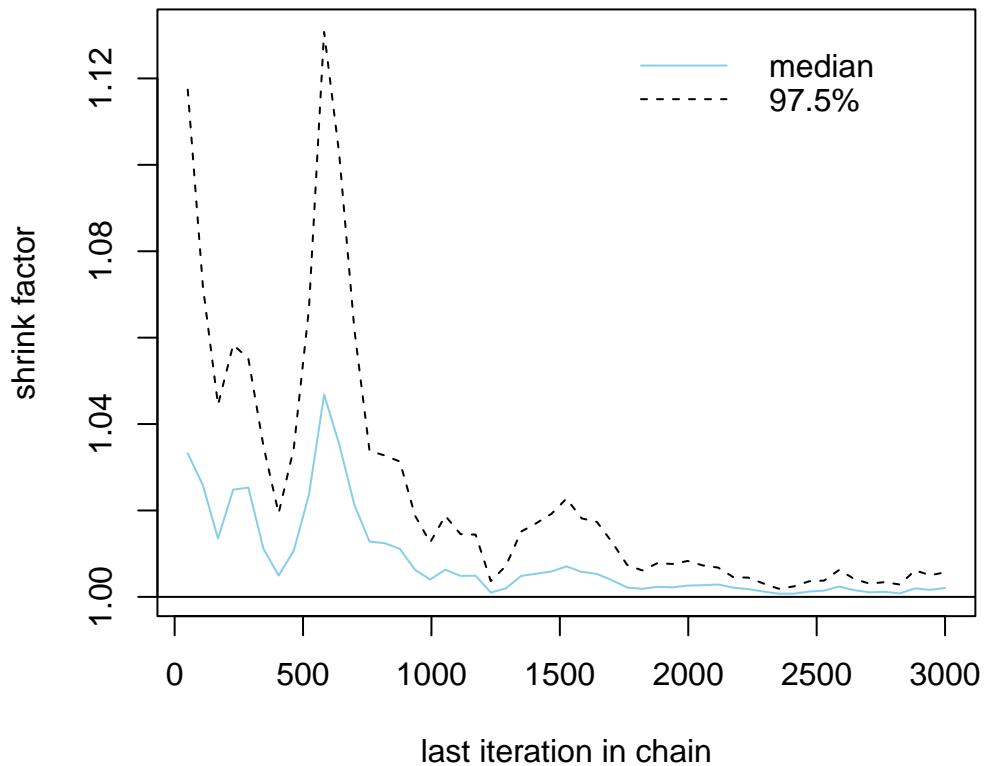

Supplement: Supplementary file 2 — Figure A2 [file 41599_2022_1441_MOESM2_ESM.pdf]

**a\_WillingEntranceFee**

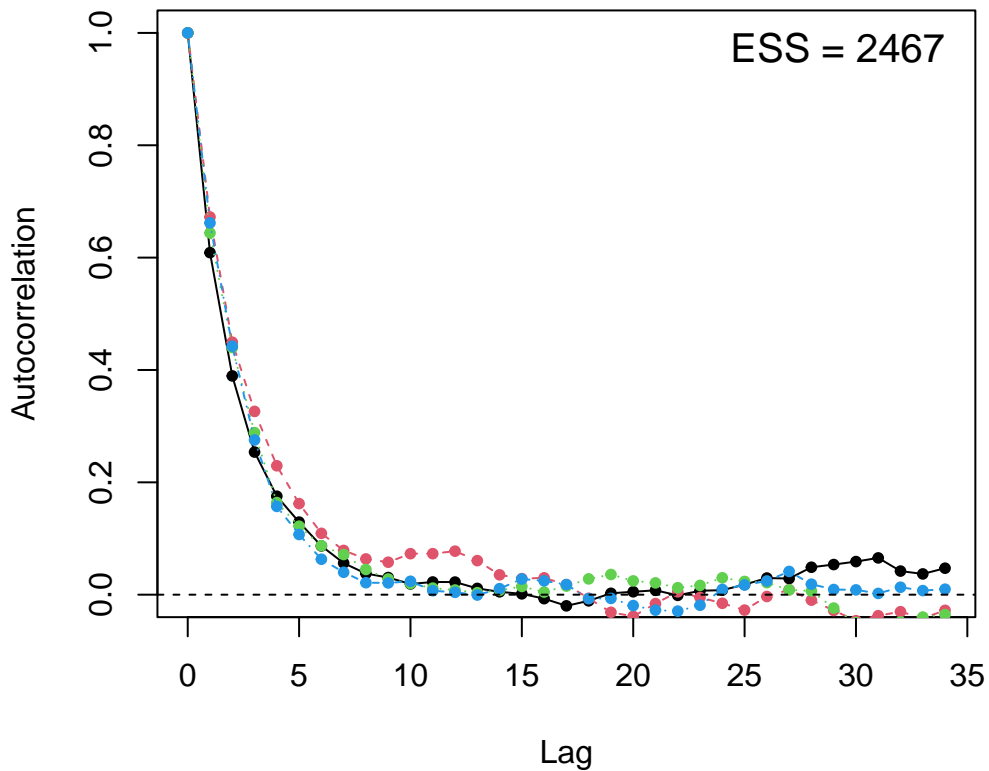

**b\_Conservation\_WillingEntranceFee**

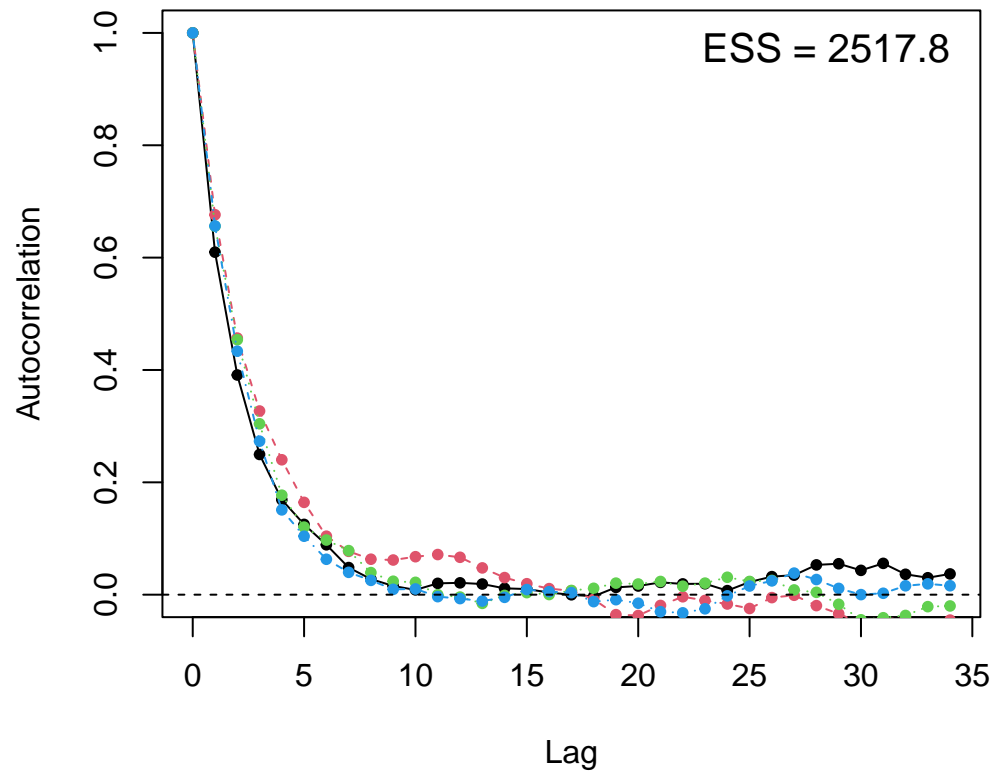

Supplement: Supplementary file 3 — Figure A3 [file 41599_2022_1441_MOESM3_ESM.pdf]

**a\_WillingDonation**

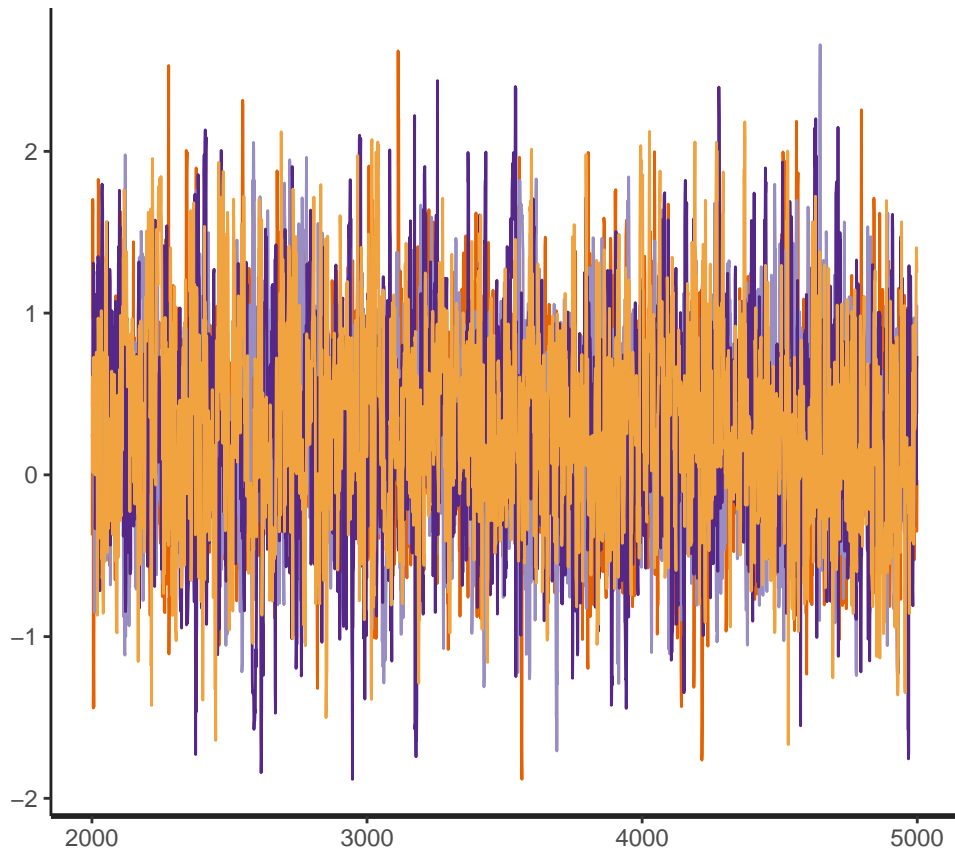

**b\_Conservation\_WillingDonation**

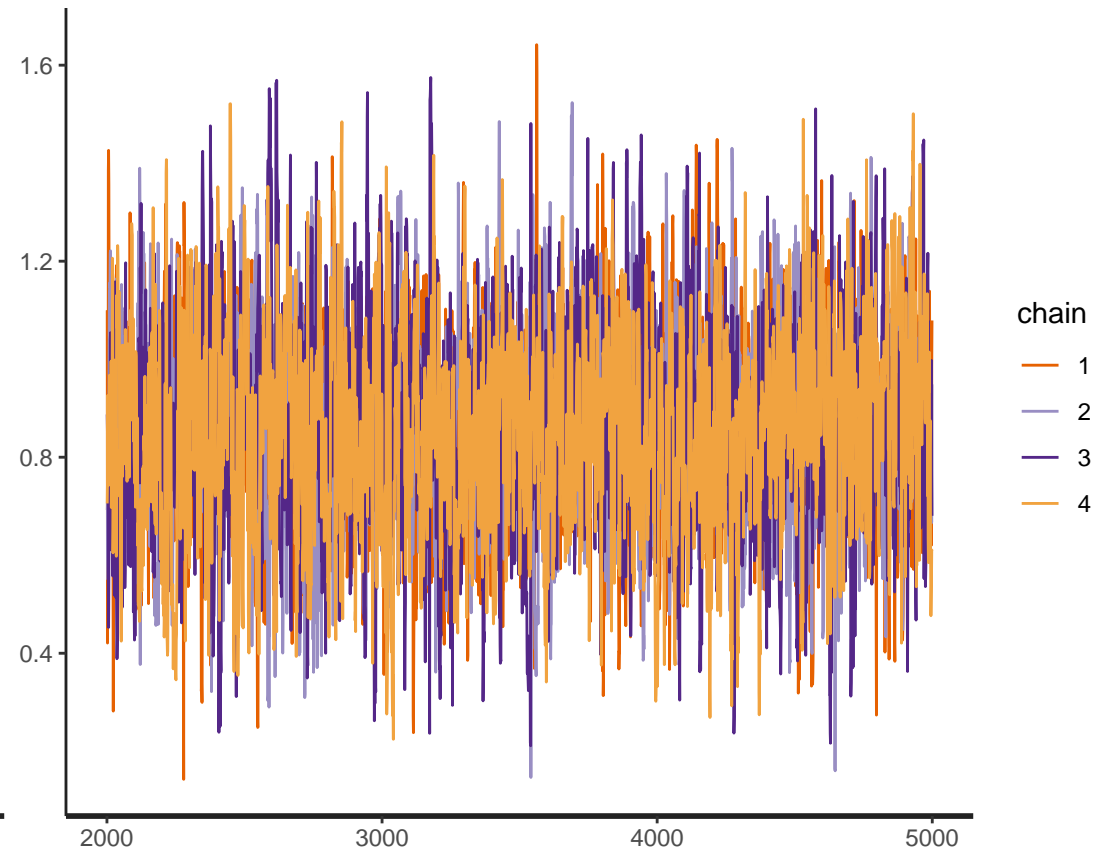

Supplement: Supplementary file 4 — Figure A4 [file 41599_2022_1441_MOESM4_ESM.pdf]

**a\_WillingDonation**

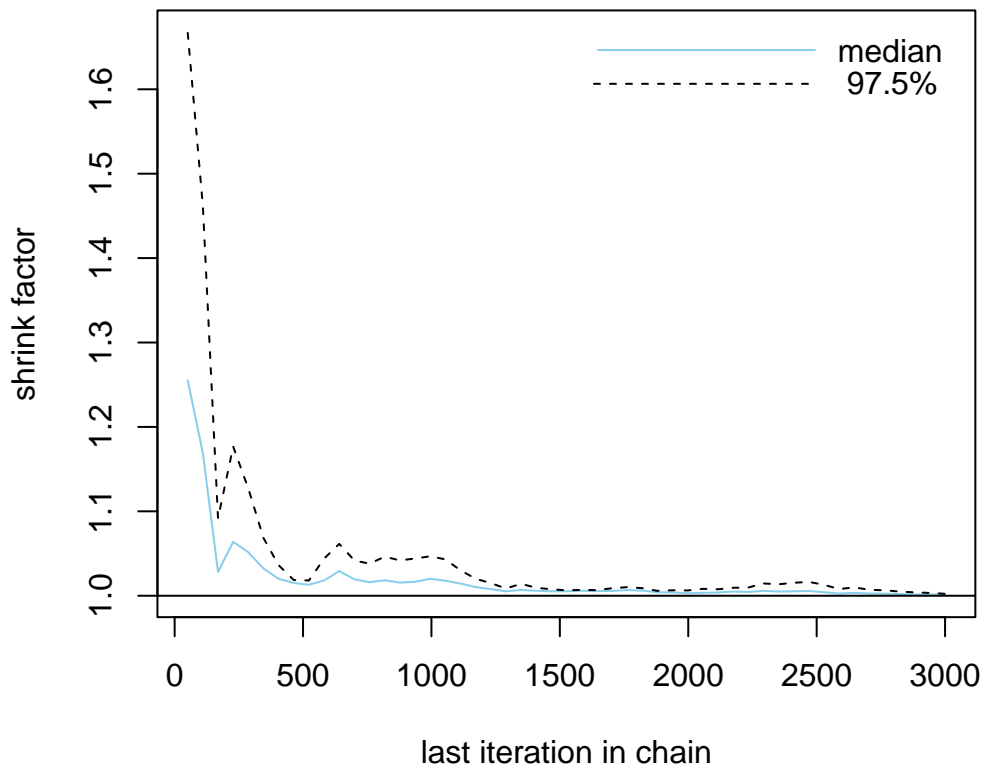

**b\_Conservation\_WillingDonation**

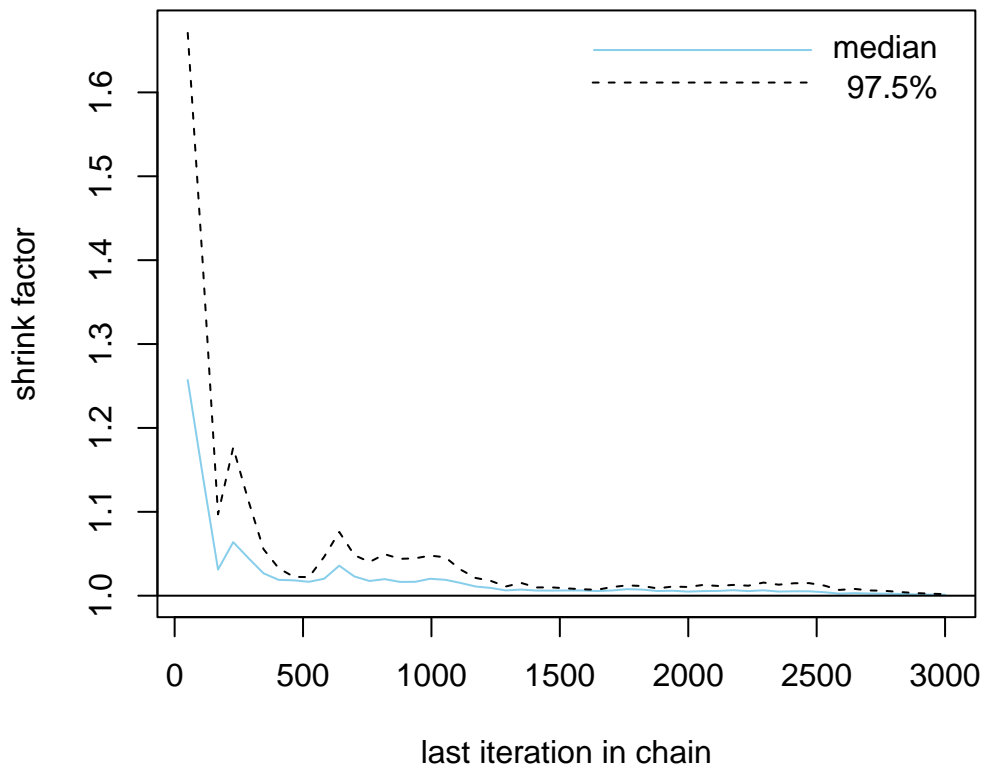

Supplement: Supplementary file 5 — Figure A5 [file 41599_2022_1441_MOESM5_ESM.pdf]

**a\_WillingDonation**

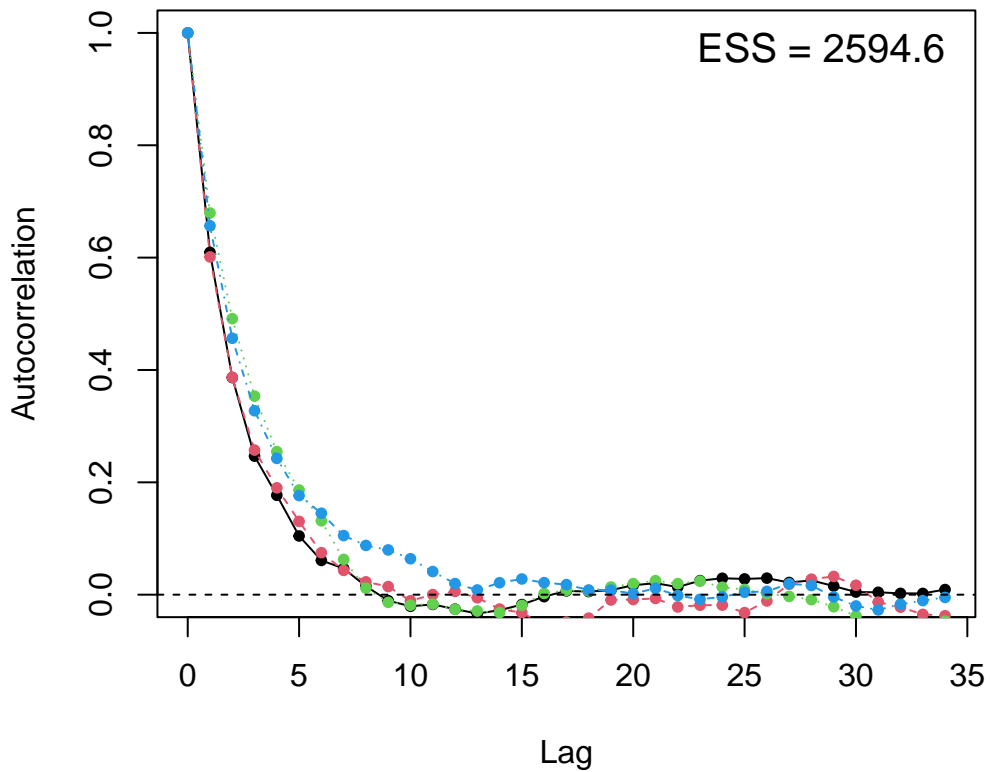

**b\_Conservation\_WillingDonation**

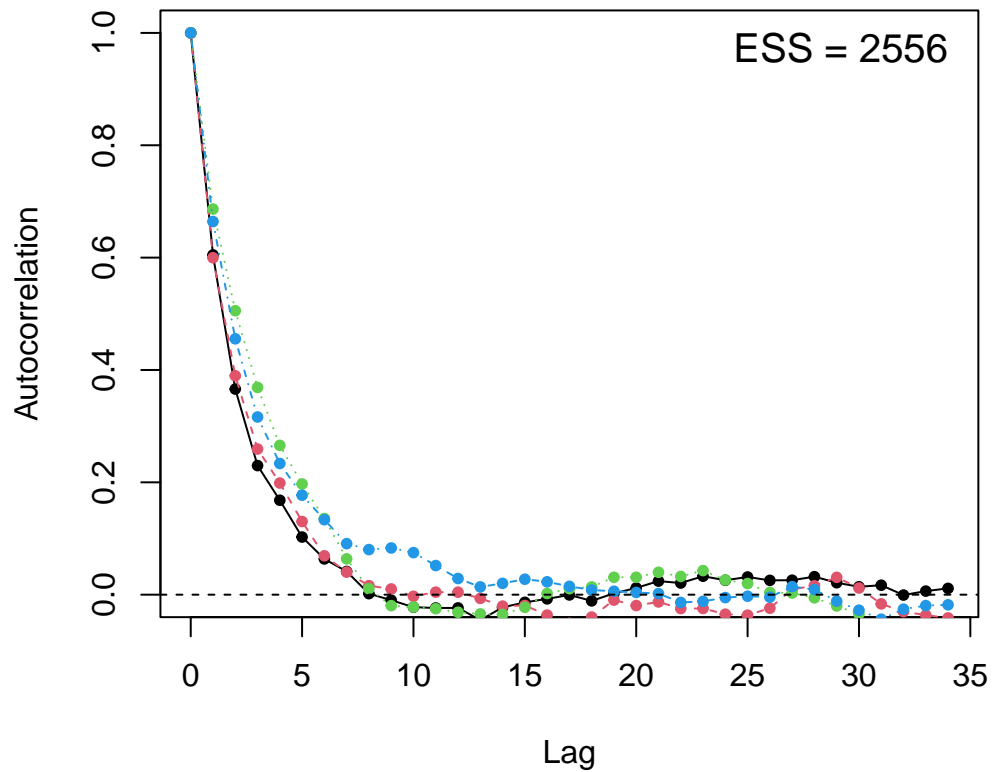

Supplement: Supplementary file 6 — Figure A6 [file 41599_2022_1441_MOESM6_ESM.pdf]
