# Supplementary material for: Building eco-surplus culture among urban residents as a novel strategy to improve finance for conservation in protected areas
Source: Humanit Soc Sci Commun. 2022 Nov 29;9(1):426. doi: 10.1057/s41599-022-01441-9 (PMC9708145; doi:10.1057/s41599-022-01441-9)
Supplement: Supplementary file 7 — Figure A7 [file 41599_2022_1441_MOESM7_ESM.pdf]

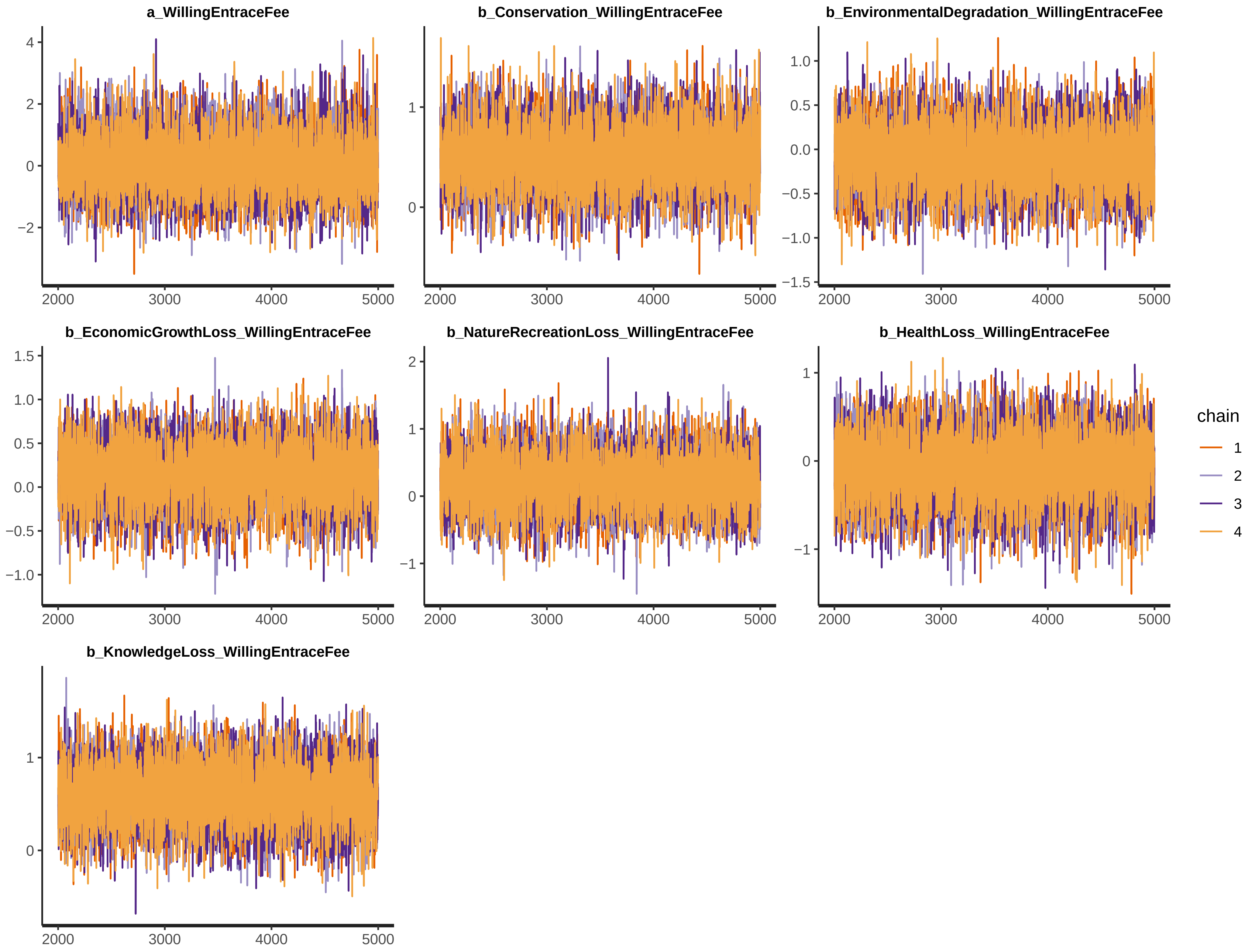
a_WillingEntraceFee
b_Conservation_WillingEntraceFee
b_EnvironmentalDegradation_WillingEntraceFee
b_EconomicGrowthLoss_WillingEntraceFee
b_NatureRecreationLoss_WillingEntraceFee
b_HealthLoss_WillingEntraceFee
b_KnowledgeLoss_WillingEntraceFee
chain
1
2
3
4
2000
3000
4000
5000
