# Supplementary material for: Building eco-surplus culture among urban residents as a novel strategy to improve finance for conservation in protected areas
Source: Humanit Soc Sci Commun. 2022 Nov 29;9(1):426. doi: 10.1057/s41599-022-01441-9 (PMC9708145; doi:10.1057/s41599-022-01441-9)
Supplement: Supplementary file 9 — Figure A9 [file 41599_2022_1441_MOESM9_ESM.pdf]

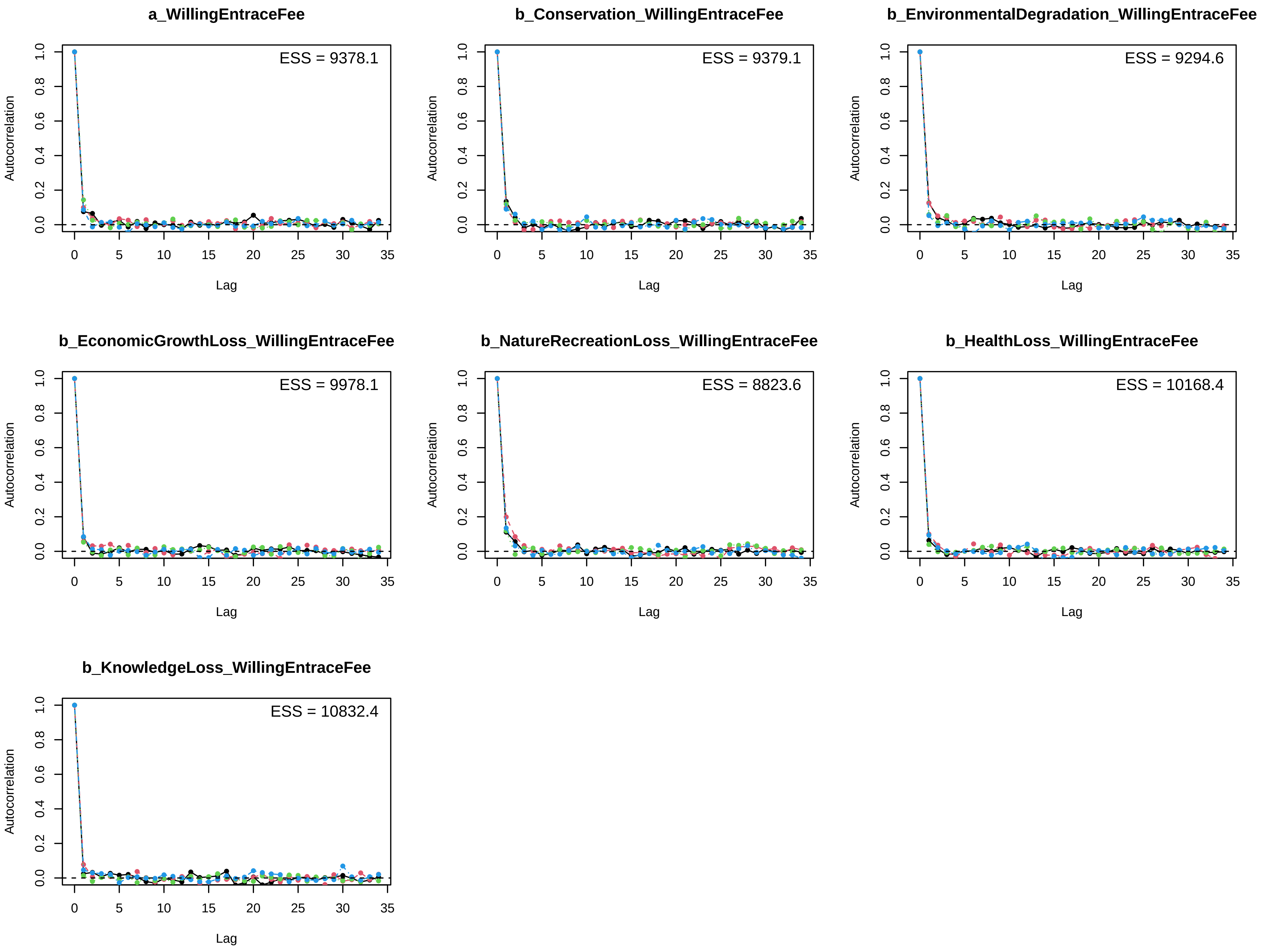
a_WillingEntraceFee
ESS = 9378.1
b_Conservation_WillingEntraceFee
ESS = 9379.1
b_EnvironmentalDegradation_WillingEntraceFee
ESS = 9294.6
b_EconomicGrowthLoss_WillingEntraceFee
ESS = 9978.1
b_NatureRecreationLoss_WillingEntraceFee
ESS = 8823.6
b_HealthLoss_WillingEntraceFee
ESS = 10168.4
b_KnowledgeLoss_WillingEntraceFee
ESS = 10832.4
Autocorrelation
Lag
0.0
0.2
0.4
0.6
0.8
1.0
0
5
10
15
20
25
30
35
