# Supplementary material for: Building eco-surplus culture among urban residents as a novel strategy to improve finance for conservation in protected areas
Source: Humanit Soc Sci Commun. 2022 Nov 29;9(1):426. doi: 10.1057/s41599-022-01441-9 (PMC9708145; doi:10.1057/s41599-022-01441-9)
Supplement: Supplementary file 10 — Figure A10 [file 41599_2022_1441_MOESM10_ESM.pdf]

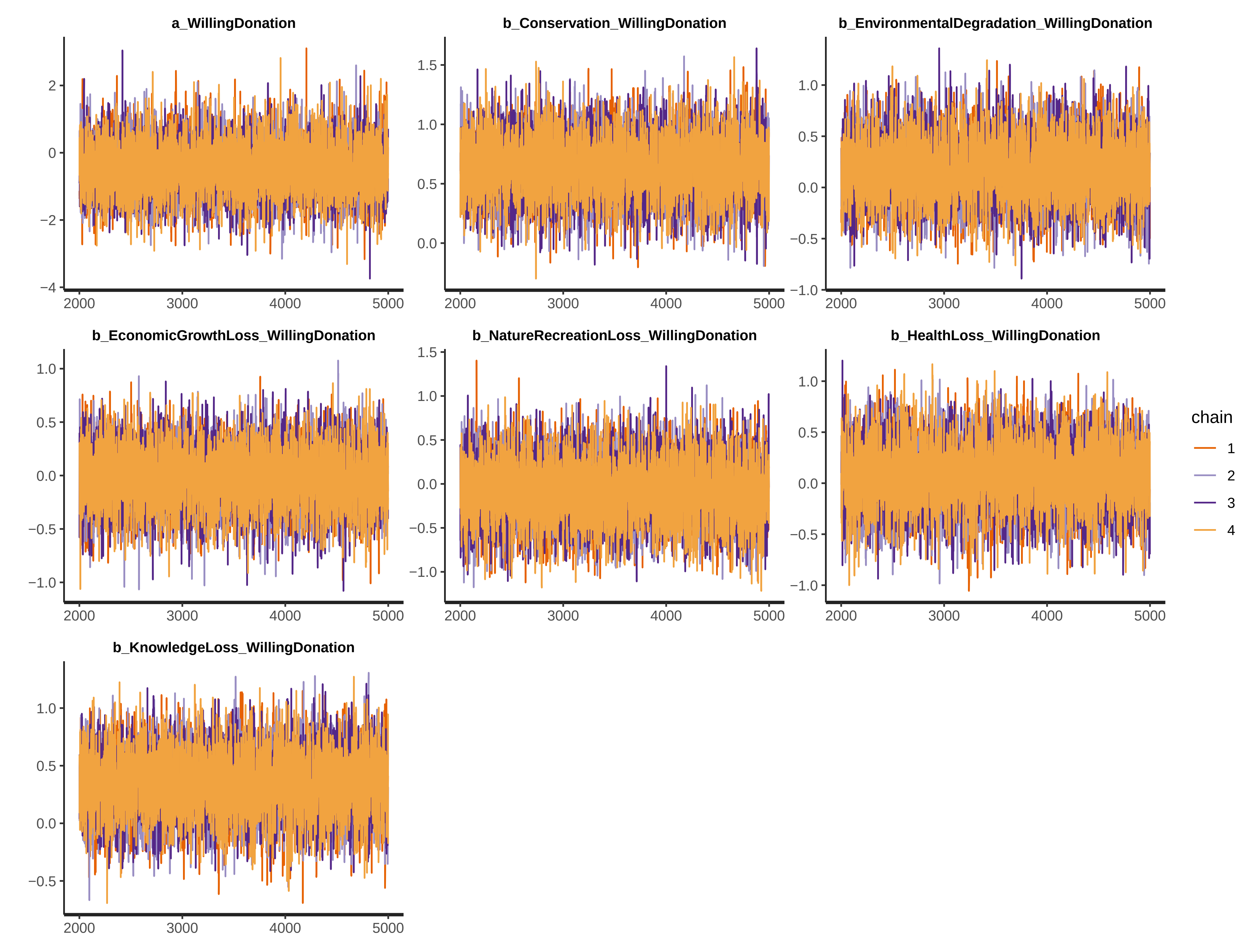

a_WillingDonation
b_Conservation_WillingDonation
b_EnvironmentalDegradation_WillingDonation
b_EconomicGrowthLoss_WillingDonation
b_NatureRecreationLoss_WillingDonation
b_HealthLoss_WillingDonation
b_KnowledgeLoss_WillingDonation
chain
1
2
3
4
2000
3000
4000
5000
