# Supplementary material for: Building eco-surplus culture among urban residents as a novel strategy to improve finance for conservation in protected areas
Source: Humanit Soc Sci Commun. 2022 Nov 29;9(1):426. doi: 10.1057/s41599-022-01441-9 (PMC9708145; doi:10.1057/s41599-022-01441-9)
Supplement: Supplementary file 11 — Figure A11 [file 41599_2022_1441_MOESM11_ESM.pdf]

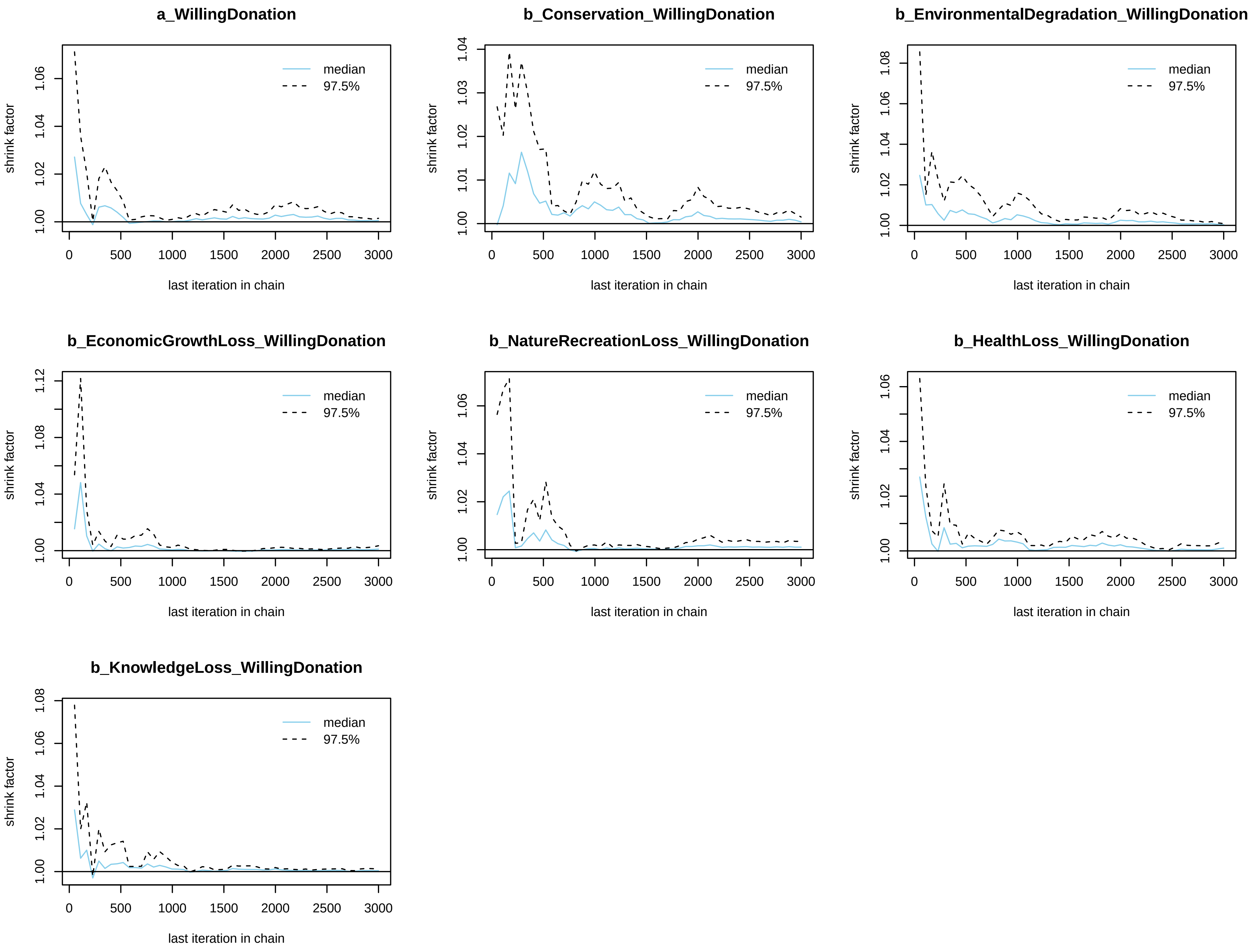
a_WillingDonation
b_Conservation_WillingDonation
b_EnvironmentalDegradation_WillingDonation
b_EconomicGrowthLoss_WillingDonation
b_NatureRecreationLoss_WillingDonation
b_HealthLoss_WillingDonation
b_KnowledgeLoss_WillingDonation
shrink factor
last iteration in chain
median
97.5%
0
500
1000
1500
2000
2500
3000
1.00
1.01
1.02
1.03
1.04
1.06
1.08
1.12
