# Supplementary material for: Building eco-surplus culture among urban residents as a novel strategy to improve finance for conservation in protected areas
Source: Humanit Soc Sci Commun. 2022 Nov 29;9(1):426. doi: 10.1057/s41599-022-01441-9 (PMC9708145; doi:10.1057/s41599-022-01441-9)
Supplement: Supplementary file 12 — Figure A12 [file 41599_2022_1441_MOESM12_ESM.pdf]

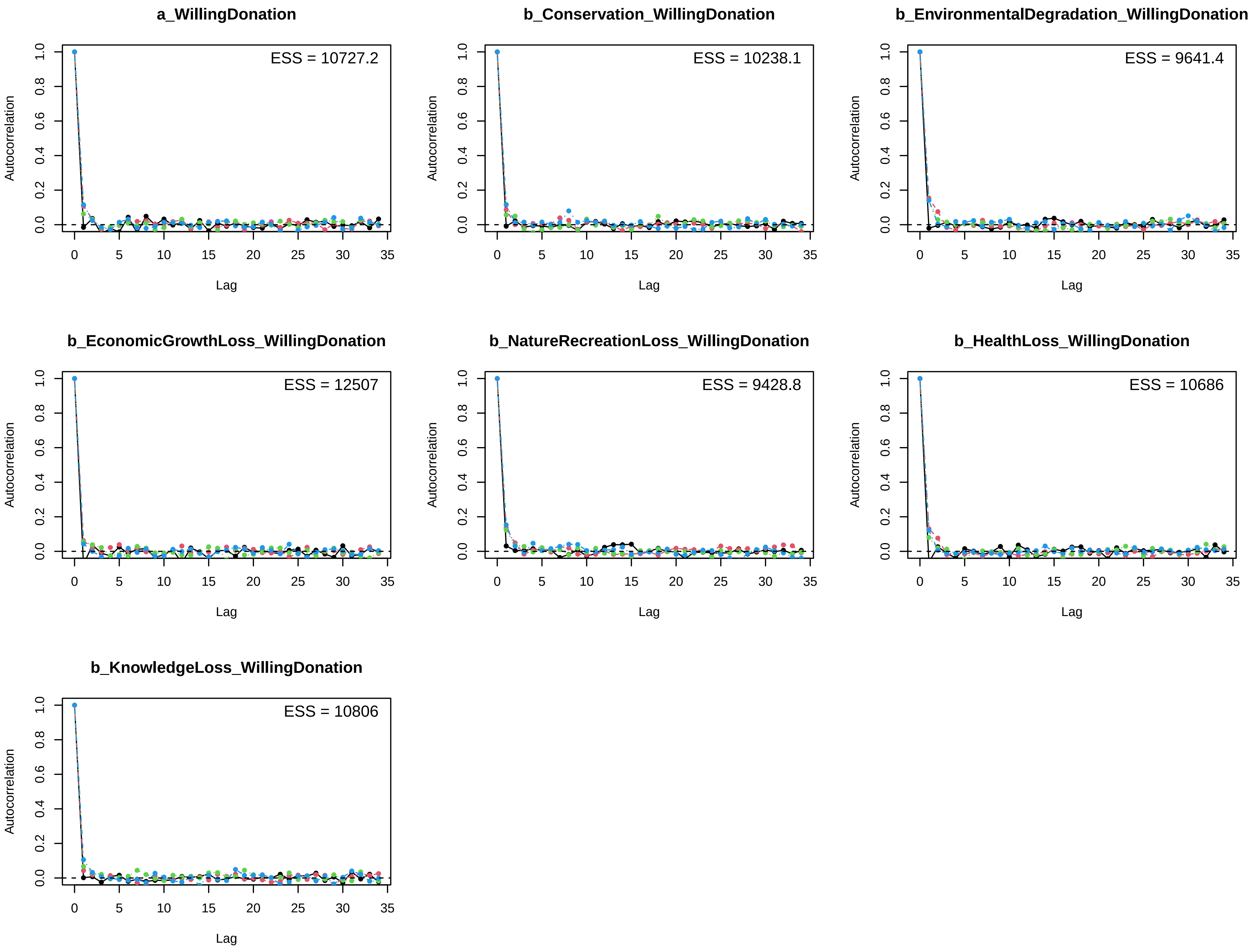
a_WillingDonation
ESS = 10727.2
b_Conservation_WillingDonation
ESS = 10238.1
b_EnvironmentalDegradation_WillingDonation
ESS = 9641.4
b_EconomicGrowthLoss_WillingDonation
ESS = 12507
b_NatureRecreationLoss_WillingDonation
ESS = 9428.8
b_HealthLoss_WillingDonation
ESS = 10686
b_KnowledgeLoss_WillingDonation
ESS = 10806
Autocorrelation
Lag
0.0
0.2
0.4
0.6
0.8
1.0
0
5
10
15
20
25
30
35
